# Synthetic X‑ray‑driven tracking and control of miniature medical devices

**DOI:** 10.1038/s42256-026-01190-3

**Published:** 2026-02-23

**Authors:** Chunxiang Wang, Wenbin Kang, Mengmeng Sun, Hongchuan Zhang, Chong Hong, Sinan Ozgun Demir, Halim Ugurlu, Kun Hao, Zemin Liu, Tianlu Wang, Metin Sitti

**Affiliations:** 1 Physical Intelligence Department, Max Planck Institute for Intelligent Systems, Stuttgart, Germany; 2 Department of Information Technology and Electrical Engineering, ETH Zurich, Zurich, Switzerland; 3 Department of Mechanical Engineering, City University of Hong Kong, Hong Kong, China; 4 Department of Biomedical Engineering, National University of Singapore, Singapore, Singapore; 5 Zentrum für Radiologie Heilbronn, Heilbronn, Germany; 6 Department of Pharmacology, Shandong University, Jinan, China; 7 Department of Mechanical Engineering, University of Hawaiʻi at Mānoa, Honolulu, HI USA; 8 School of Medicine and College of Engineering, Koç University, Istanbul, Turkey

**Keywords:** Electrical and electronic engineering, Mechanical engineering

## Abstract

The clinical translation of miniature medical devices (MMDs) for minimally invasive surgery promises transformative advances in biomedical engineering, offering enhanced precision, reduced patient trauma and faster recovery times. However, their effective deployment in complex anatomies under real-time X-ray guidance—a widely used surgical imaging modality—presents challenges such as low imaging quality and difficulties of spatial MMD control. Manual identification and operation are labour intensive and error prone. Meanwhile, deep learning-based automation is limited by the scarcity of annotated X-ray datasets of MMDs owing to costly data collection, laborious annotation and privacy constraints. Here we introduce MicroSyn-X, a framework for training computer vision models to enable robotic teleoperation of MMDs using synthesized high-fidelity, pixel-accurate, auto-labelled and domain-randomized X-ray images, eliminating manual data curation. Integrating MicroSyn-X into a teleoperated robotic system enables real-time localization and navigation of magnetic soft and magnetic liquid MMDs within both ex vivo and dynamic in vivo environments, demonstrating robustness under challenging imaging conditions of low contrast, high noise and occlusion. With these promises, we open source the X-ray MMD dataset to enable benchmarking. Addressing data scarcity and enabling real-time robotic navigation, this work advances MMD-assisted minimally invasive surgery towards next-generation precision interventions.

## Abstract (web summary)

Wang et al. introduce MicroSyn-X, a synthetic X-ray data generation framework that overcomes data scarcity in miniature medical devices, enabling robust deep learning-based tracking and real-time robotic navigation in challenging surgical settings.

## Main

The integration of miniature medical devices (MMDs), ranging from miniature robots^1–3^ to implantable biosensors^4–6^, into minimally invasive surgical practices is transformative in biomedical engineering^7,8^. Specifically, small-scale devices actuated by external fields can navigate through enclosed spaces challenging for conventional tethered tools^7,8^ and offer functionalities such as drug delivery^9–11^ and physiological property sensing^12–14^. To translate these innovations towards clinical applicability, safe and effective MMD deployment is essential, requiring real-time medical imaging to continuously monitor the operation in non-transparent biological environments^15^. Among available medical imaging modalities^16–32^ (Extended Data Tables 1 and 2), X-ray fluoroscopy is widely used in surgeries given its deep tissue penetration, high resolution, near real-time imaging and expansive visualization window^33,34^. However, fluoroscopic image-guided operation in dynamic, cluttered anatomical environments remains labour intensive, leading to operator fatigue and reduced precision^35–37^. Limitations persist in manual object identification from overlapping anatomical features^38,39^ and precise manual adjustments of tools^37,40–42^, underscoring the imperative for robotic systems with automated object tracking to improve procedural efficiency and alleviate human effort.

**Fig. 1:**
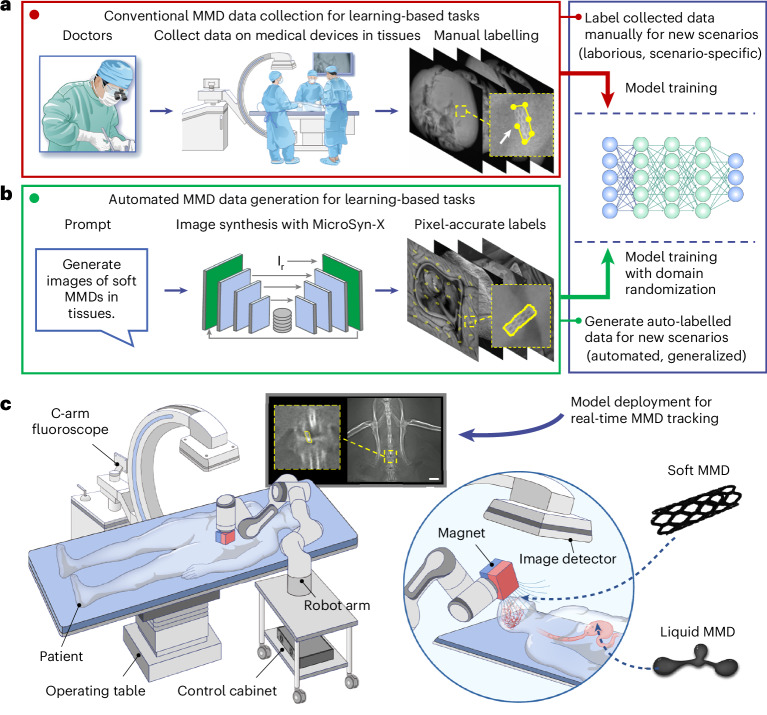
Overall concept of MicroSyn-X. **a**, The conventional data collection of MMDs for learning-based tasks. Clinicians perform labour-intensive image collection and annotation in real tissues, requiring repeated efforts for new MMDs or anatomical targets. **b**, The automated model training with MicroSyn-X. MicroSyn-X automates domain-adapted synthetic image generation, eliminating manual labelling and enabling scalable training across diverse MMD–tissue scenarios. MMD images are denoted by ${{\rm{I}}}_{{\rm{r}}}$. **c**, A robotic platform for magnetic MMD manipulation. A robotic arm with an actuating magnet remotely guides MMDs, including stent-structured and shape-morphable liquid MMDs, through anatomical barriers. Real-time C-arm fluoroscopy visualizes the MMD, while the machine learning model trained with MicroSyn-X provides guidance feedback. The image is from an in vivo animal experiment with a 5 mm scale bar.

Deep learning excels at medical image analysis, particularly in complex environments challenging for conventional image-processing techniques using handcrafted features^43–46^. However, its performance relies on large, high-quality annotated datasets. Insufficient data causes poor generalization, resulting in deceptively high training accuracy and performance degradation on unseen clinical settings^47^. Furthermore, data scarcity is common in medicine owing to the challenges of data collection and annotation^48,49^ (Fig. 1a). Data collection is hindered by the need for specialized imaging equipment to capture MMDs within tissues and stringent ethical regulations restricting public access^50,51^. Meanwhile, annotation demands meticulous manual effort, such as selecting precise bounding points, which is laborious and prone to human error due to cognitive fatigue^48,49^.

Synthetic data offer a transformative solution to data scarcity by leveraging generative models or simulations to create artificial datasets that mimic the structural properties of real-world data^50–52^. This further enables the creation of diverse datasets that mitigate class imbalance, where rare conditions or underrepresented objects hinder model generalization^48^. Current applications have demonstrated their clinical utility^49^, with well-validated generation quality and enhanced downstream model performance in surgical scene synthesis for organ segmentation^53–57^ and depth estimation^58–60^, privacy-preserving X-ray image generation for pathological analysis^61–64^ and disease image synthesis^65–69^.

Small-scale medical devices present unique challenges for image-guided deployment. Unlike macroscopic features, these tiny components appear as low-contrast, noisy entities within cluttered anatomical scenes, easily occluded by surrounding tissues and manipulation tools^70,71^. Furthermore, MMD deployment requires real-time tracking and integration into robotic systems to ensure precision and robustness under clinical constraints^72^, such as poor imaging conditions and dynamic anatomical variability. The utilization of state-of-the-art computer vision (CV) models in MMD-relevant scenarios is impeded by the absence of publicly accessible datasets and generative models tailored for MMDs. While general-purpose models such as the Segment Anything Model^73,74^ and vision language models^75,76^ excel in broad CV tasks, they require fine-tuning with domain-specific data in specific medical contexts^77–80^. Likewise, self-supervised learning for label-free model training minimizes annotation but still demands high-quality datasets^81–83^. In addition, current robotic systems for MMD deployment exhibit limitations, such as manual object identification^3,9,14,42^, experiments within simplified phantoms instead of realistic clinical environments^84–86^ and focus on macroscopic devices with good visibility^51,87,88^.

To address the challenges of tracking and deploying micro- and millimetre-scale MMDs in complex anatomical environments, we propose MicroSyn-X, a framework that synthesizes X-ray MMD images for training CV models, and develop a teleoperated robotic system achieving vision-based MMD control using these trained models. Unlike conventional approaches of recursively curating and annotating MMD data (Fig. 1a), MicroSyn-X enables end-to-end synthesis of high-fidelity, pixel-accurately labelled MMD data (Fig. 1b). Guided by user prompts, it controllably generates images of MMDs inside diverse environments to mimic clinical scenarios with domain randomization, where scene properties, such as MMD appearances and background complexity, are randomized. This approach enables rapid adaptability to new applications, addressing data scarcity while eliminating manual labelling. Furthermore, the synthesized data directly trains CV models to deploy on robotic systems, enabling precise MMD navigation despite low contrast, occlusion and imaging noise under clinical X-ray fluoroscopy (Fig. 1c), relieving users of labour-intensive operations. We demonstrate real-time deployment and tracking of two representative MMDs, including magnetically actuated soft MMDs^3,9^ and shape-adaptable liquid MMDs^89^ in ex vivo and in vivo environments, validating the framework’s effectiveness. Notably, we open-source the X-ray MMD dataset, facilitating benchmarking and democratizing research in MMDs. Bridging the data gap and enabling precise MMD control, this work advances the feasibility of MMD-assisted minimally invasive surgery under clinical X-ray imaging, marking a critical step towards next-generation precision interventions.

## Results

### Workflow of MicroSyn-X

**Fig. 2:**
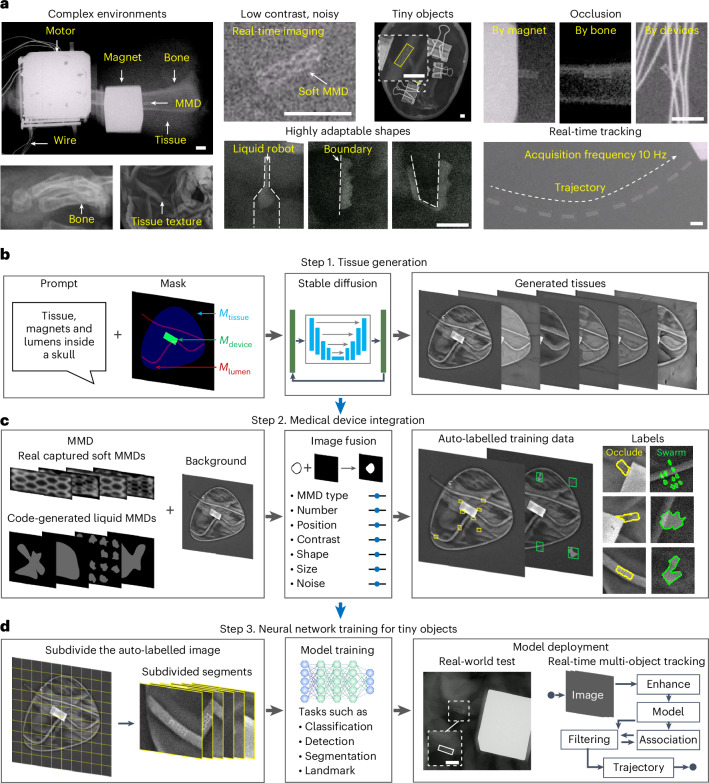
Workflow of MicroSyn-X. **a**, The clinical challenges of MMD perception under real-time X-ray imaging. **b**, The controlled tissue generation process. Stable diffusion creates high-fidelity tissue images from user-defined masks and prompts. **c**, The integration of medical devices with tissues. Captured or generated MMD images are seamlessly integrated into the background with flexible parameters, ensuring pixel-accurate labelling. **d**, A schematic of neural network training for MMD tracking. MMD–tissue images are subdivided to improve precision and efficiency in tiny object localization. The model, trained on synthetic data, is then deployed for real-world MMD tracking.

The challenges of tracking MMDs under clinical fluoroscopy are summarized in Fig. 2a, including complex imaging environments, tiny objects in low-contrast and noisy scenes, occlusion and adaptive MMD shapes. X-ray imaging leverages differential attenuation of X-rays through materials with varying density and atomic composition, presenting constraints in clinical settings^33,34^. First, all anatomical structures are captured within the field of view, creating visual distractions that obscure MMDs with inherently poor visibility, requiring operators to manually search objects. Occlusion further complicates localization, as dense tissues such as bone or metallic components can block MMDs in two-dimensional (2D) projections^70,71^. Moreover, imaging noise degrades clarity, particularly under low milliampere second (mAs) fluoroscopic settings^90^. Specifically, the shape-deformable liquid MMDs enables the navigation of confined spaces but requires CV models to handle continuously changing shapes^89,91,92^.

We present MicroSyn-X, a modular, label-free end-to-end synthetic data generation pipeline for clinical CV model deployment, structured into three stages for MMD tracking. First, background generation leverages a diffusion model to synthesize surgical scenes with anatomically realistic tissue textures and operation tools. Second, magnetic MMDs, captured from real-world scenarios or algorithmic generation, are programmatically overlaid onto synthetic backgrounds. This process incorporates domain randomization, adjusting parameters such as contrast and shape, while generating pixel-accurate labels. This step eliminates manual annotation, a critical bottleneck in medical CV tasks. Finally, the synthetic dataset is used to train a downstream CV model for tasks such as detection and segmentation. The trained models are integrated into a real-time object tracking framework for soft and liquid MMDs in physiological environments. This framework offers a fast and scalable solution to train CV models for MMD navigation.

The framework firstly employs a pix2pix stable diffusion model^93^ to synthesize surgical scenes utilizing user prompts and mask inputs, as shown in Fig. 2b. The input mask image, composed of three channels (tissue area ${M}_{\mathrm{tissue}}$, metallic device area ${M}_{\mathrm{device}}$ and contrast agent-filled lumen area ${M}_{\mathrm{lumen}}$), enables precise control over anatomical structures and device placement. Users can customize the shape, position and brightness of these regions while providing prompts to specify scene composition. This hybrid approach, combining conditional diffusion with spatial guidance, generates backgrounds replicating anatomical variability, such as differences in organ geometry or tissue density, critical for training robust CV models.

MMD integration (Fig. 2c) further merges MMD images and tissue via an add-weighted strategy (see ‘MMD data preparation and integration’ in Methods). This approach mimics the X-ray imaging mechanism, where the MMD modulates the X-ray signals of tissues, creating overlapping attenuation in a 2D projection^33^. MMD images are obtained by capturing static devices on clean backgrounds or generating deformable liquid MMD shapes using parametric spline curves to mimic dynamic shape changes (Supplementary Fig. 1 and ‘Mask generation with spline curves’ in Methods). The MMD is pasted with a known mask, automatically generating labels (class and bounding points) without manual annotation. To reflect clinical realism, Poisson, Gaussian and pepper noise are injected to simulate imaging noise, while occlusion is modelled by positioning the MMD beneath the ${M}_{\mathrm{device}}$ area. This workflow ensures synthetic data captures heterogeneous tissue–MMD interactions and contrast variations, offering computational efficiency and scalability for large datasets.

To train a CV model with strong generalization capabilities and minimize the sim-to-real gap, domain randomization is implemented in two approaches (Fig. 2b,c). First, background randomization introduces variability in anatomical and imaging conditions by altering tissue type, brightness, shape and position by adjusting user prompt and ${M}_{\mathrm{tissue}}$; device morphology (${M}_{\mathrm{device}}$) parameters such as shape, position and brightness; lumen structures (${M}_{\mathrm{lumen}}$) with randomized shape and contrast; and controlled noise levels. Second, MMD-specific attributes are randomized, including type, quantity, position, shape (via geometrical transformation) and contrast. This strategy ensures a broad data distribution, forcing the CV model to prioritize invariant features (such as MMD contours) over spurious correlations (such as texture-specific cues), a principle validated in domain generalization studies^48,51^.

For localizing MMDs in large, cluttered images, where small objects risk being drowned out by irrelevant background features, a patch-based strategy is utilized for CV model training and inference (Fig. 2d and Supplementary Fig. 2). Images are subdivided into smaller patches for model training and inference, aligning with evidence that deep neural networks excel at capturing fine-grained details when trained on region-specific data^94,95^. By prioritizing local features over global context, the method mitigates challenges of low contrast and noise and improves model generalization, and enables scalable training on memory-constrained hardware, as smaller tiles fit within graphics processing unit (GPU) memory limits while maintaining high-resolution analysis. Last, the trained models are integrated into a multi-object tracking framework, enabling continuous MMD localization.

### Tissue generation and open-source X-ray MMD dataset

Synthetic tissue background generation is critical for training robust CV models considering the heterogeneous tissue textures, dynamic occlusions and variable noise of real scenarios. It is difficult to capture all such variations with manual data collection, whereas synthetic data provide an efficient and cost-effective alternative. Diffusion models have proven effective for generating realistic medical images^49,52,65^ owing to their training stability and fine detail synthesis^56,62,63,68^. We employed a pix2pix diffusion model^93^ to generate X-ray images with minimal manual efforts (Supplementary Fig. 3 and ‘Diffusion model training and inference’ in Methods); existing X-ray datasets, such as the small-mammal anatomical dataset^96^, can also be incorporated.

**Fig. 3:**
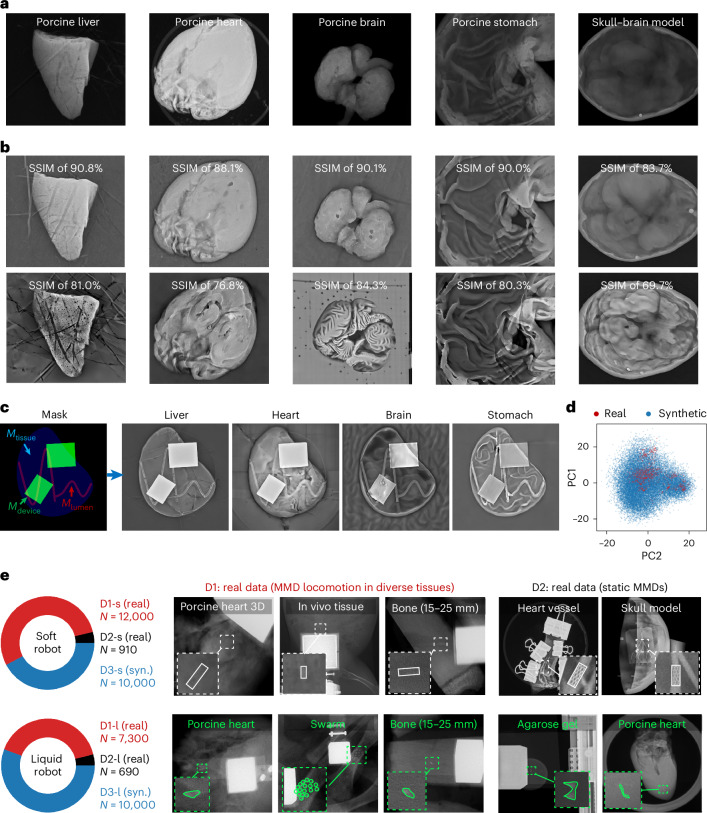
Domain randomization of synthetic tissue images and open-sourced MMD X-ray dataset. **a**, Real tissue images under X-ray imaging. **b**, The generation of precisely matched synthetic tissues with randomized and enhanced textures. **c**, Image generation with mask-guided conditioning and prompts. Randomization of masks and prompts substantially expands the dataset. **d**, A domain comparison of real and synthetic tissues. Features from 1,140 real and 24,803 synthetic tissue images, extracted using Inception V3, are visualized via PCA. **e**, An overview of the open-source MMD dataset under X-ray imaging. Dataset 1 (D1) contains real-time recordings and annotations of MMD locomotion. Dataset 2 (D2) includes static MMD images captured under various voltage and current conditions. Dataset 3 (D3) provides synthetic images with corresponding segmentation labels. The suffixes ‘-s’ and ‘-l’ denote the soft and liquid MMDs, respectively. Source data.

The generation results demonstrate that domain randomization effectively enhances tissue heterogeneity. When randomizing parameters such as diffusion steps and prompt guidance weights the framework produced high-fidelity backgrounds with a structural similarity index (SSIM)^97^ ranging from 0.65 to 0.91 compared with real images (Fig. 3a,b). Programmatically varying mask shapes and prompts further enables diverse anatomical textures within controlled boundaries, alongside customized metallic devices and lumens (Fig. 3c). Domain analysis validates the expanded diversity of synthetic data: Inception V3 feature extraction^98^ and principal component analysis (PCA)^99^ reveal a broader distribution than real data, covering underrepresented regions (Fig. 3d). As generation quality directly influences downstream model performance, quality control procedures are detailed in the ‘Diffusion model training and inference’ in Methods.

**Extended Data Fig. 1:**
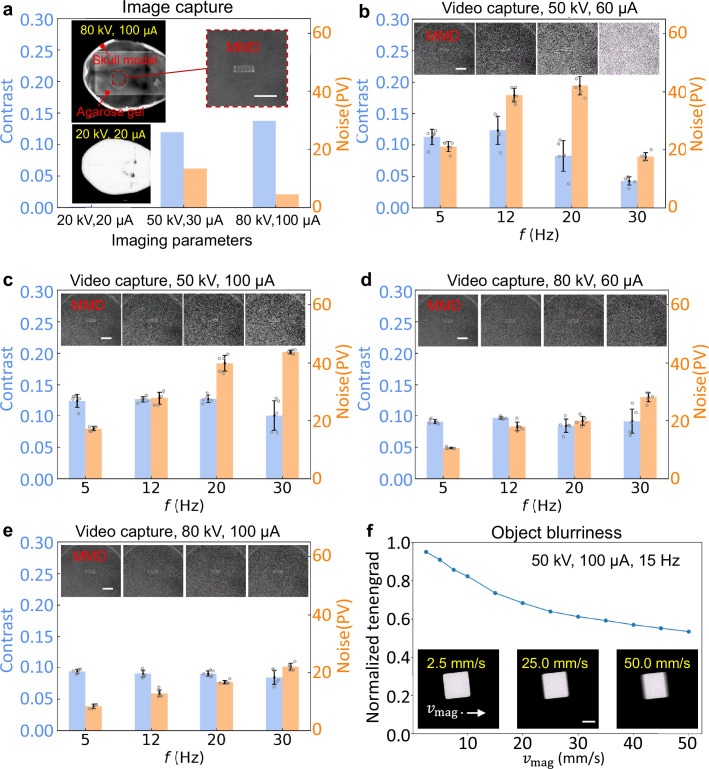
Effect of imaging parameters on image quality. **a**. Impact of X-ray voltage and current on image contrast and noise in static capture mode. **b–e**. Influence of voltage, current, and frame capture rate ($f$) on contrast and noise in continuous capture mode. Data are presented as mean values $\pm$ standard deviation. Error bars denote standard deviation across five images. **f**. Quantification of motion-induced blurriness as a function of magnet translation speed (${v}_{{\rm{mag}}}$) under continuous capture. Blurriness is assessed via the normalized Tenengrad sharpness metric, where lower values indicate increased motion blur. All experiments were performed using the XPERT 80 imaging system (KUBTEC Scientific). In all figures, scale bars represent 5 mm. Source data.

This X-ray MMD dataset is open source, featuring stent-structured soft MMDs^3,9^ and shape-morphing ferrofluid MMDs^89^ (Fig. 3e and Supplementary Fig. 4). The dataset is composed of real and synthetic domains. The real domain comprises dynamic (D1) and static (D2) subsets: D1 captures real-time MMD locomotion across diverse tissues, while D2 contains static MMDs under varying imaging conditions (Extended Data Fig. 1 and ‘Dataset preparation’ in Methods). The static subset enables quantitative evaluation of imaging parameter effects on model performance, while dynamic datasets facilitate testing tracking algorithms. The synthetic dataset (D3) was created by MicroSyn-X. This publicly available dataset provides a foundation for training and benchmarking CV models on MMD data, enabling systematic comparison of different architectures and training strategies.

### Evaluation of MicroSyn-X

**Extended Data Fig. 2:**
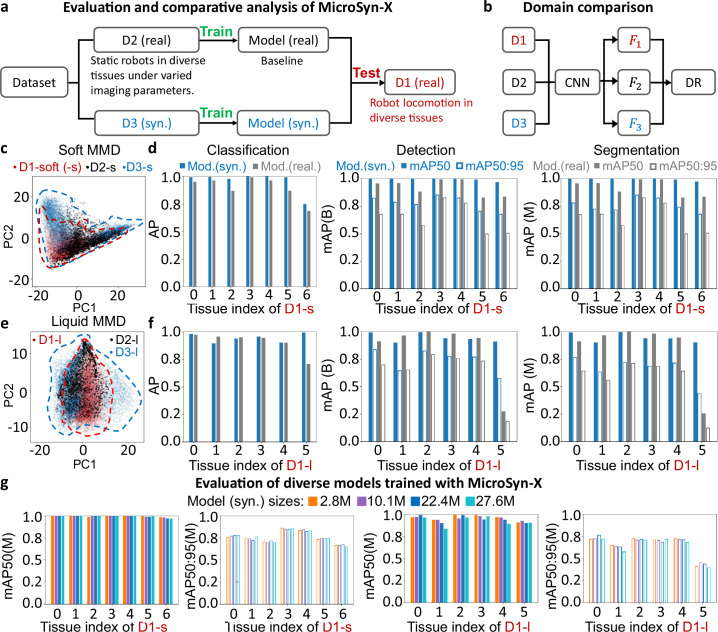
Evaluation of MicroSyn-X. **a**. Schematic of the evaluation process. **b**. Cross-domain feature comparison using CNN-extracted features visualized by dimensionality reduction (DR). **c**. Comparison of soft MMD datasets via PCA, with dashed lines indicating data distribution boundaries. **d**. Performance of models trained on synthetic and real soft MMD data across classification, detection, and segmentation tasks, measured by AP, mAP50, and mAP50:95. mAP(B) and mAP(M) denote bounding-box and mask mAPs, respectively. Tissue index indicates datasets: soft MMDs in porcine brain with embedded bones, porcine brain, heart, liver, stomach, heart 3D vessels, and in vivo animals. Models with a parameter size of 10.1 M is utilized. **e**. Cross-domain comparison of liquid MMD datasets. **f**. Performance comparison between models trained on synthetic and real liquid MMD data. Tissue index indicates datasets: liquid MMDs in porcine brain, heart, liver, stomach, brain or liver with embedded bones, and robot swarm under bone occlusion. **g**. Performance evaluation of models trained on synthetic data of soft and liquid MMDs, with varying model sizes (2.8 M, 10.1 M, 22.4 M, 27.6 M). Source data.

The evaluation aims to assess its ability to bridge the synthetic-to-real gap and robustness under unpredictable imaging conditions and anatomical variability. It is compared with baselines of conventional CV model training and clinical experts and validated across ex vivo tissues, in vivo experiments, multiple CV models and various imaging conditions. Unlike incomplete and expensive real data, synthetic data facilitate expanding data distributions and improving CV model adaptability. Three datasets are presented: D1 (real MMD locomotion) serves as the test set, while D2 (static MMDs under varied imaging conditions) and D3 (synthetic data) train models, respectively (model (syn.) and model (real)) (Extended Data Fig. 2a). Features extracted from D1–D3 via the model (syn.) backbone (${F}_{1}$,$\,{F}_{2}$ and ${F}_{3}$) are visualized via dimensionality reduction, and used for data distribution coverage analysis (Extended Data Fig. 2b-e and Supplementary Fig. 5). For real-time MMD localization, the YOLO11-seg class was adopted for its high accuracy and speed^100^ (‘CV model training and inference’ in Methods). Performance metrics included average precision (AP), mean AP at intersection of union (IoU) of 0.50 (mAP50) for basic localization accuracy and mAP50:95 for rigorous evaluation (‘Computation of metrics’ in Methods).

MicroSyn-X demonstrates generalization and robustness in realistic scenarios. For soft MMDs, model (syn.) outperforms model (real) in both mAP50 and mAP50:95, especially in low-contrast, high-noise environments such as dynamic in vivo environments (Extended Data Fig. 2d and Supplementary Fig. 6a). It is attributed to expanded data distribution through domain randomization while preserving realistic MMD appearance, covering edge cases impractical to collect in real-world settings. In the domain analysis of soft MMDs, synthetic data exhibits broader coverage compared with real data (Extended Data Fig. 2c and Supplementary Fig. 5), highlighting its ability to span underrepresented variations. For liquid MMDs, model (syn.) achieves comparable mAP50 to model (real) and surpasses it in stricter mAP50:95 (Extended Data Fig. 2f and Supplementary Fig. 6b). While mathematically generated spline curves introduce shape diversity, their simplified appearances lead to lower data coverage (Supplementary Fig. 5) and slightly reduce mAP50 for easy detections. However, this diversity enhances performance in complex tasks such as tracking swarms under bone occlusions, particularly during dynamic shape transitions (splitting and merging). Future improvements could focus on refining MMD fidelity (Supplementary Fig. 7) and incorporating physics-based deformation models^101^. Scalability tests further validate the effectiveness of MicroSyn-X (Extended Data Fig. 2g), where models of varying sizes (2.8 M, 10.1 M, 22.4 M and 27.6 M parameters) achieve comparable high accuracy.

**Extended Data Fig. 3:**
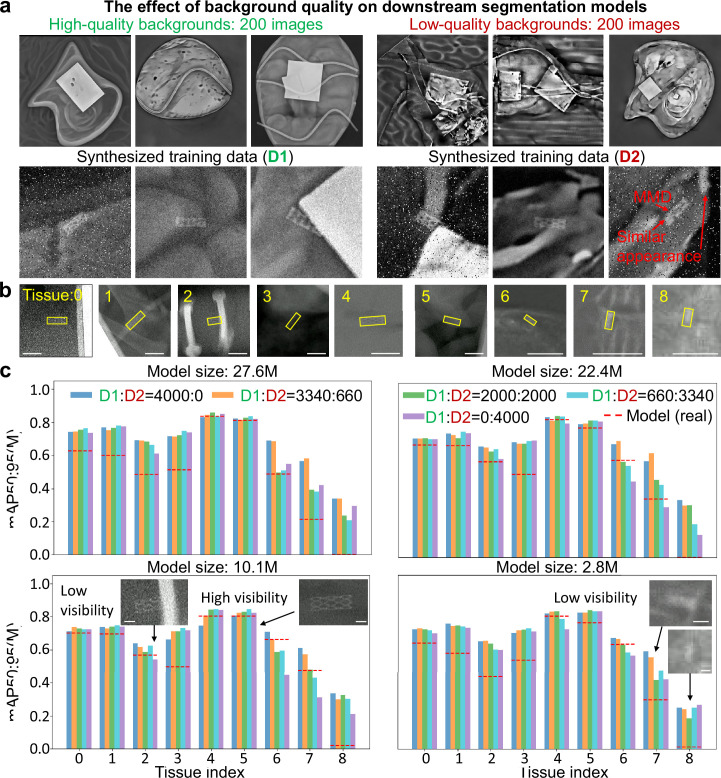
Effect of synthetic background quality on downstream segmentation models. **a**. Dataset composition. 200 high- and 200 low-quality generated tissue backgrounds were used to construct five synthetic training sets with varying proportions of high-quality backgrounds. **b**. Real-tissue test dataset. The test images include soft MMDs in porcine brain with embedded bone, porcine brain, heart, liver, stomach, heart 3D vessels, in vivo rabbit, in vivo rat, and in vivo rat spine at low magnification. Scale bars represent 5 mm. **c**. Segmentation performance across background quality ratios and model sizes. For structurally distinct targets (for example, stents) with high visibility, performance remains stable or improved, where low-quality backgrounds provide beneficial augmentation. In contrast, for low-contrast or ambiguous targets (for example, blurred white rectangles resembling background artefacts), inclusion of a modest fraction of low-quality backgrounds (ratio = 0.86) improves or maintains performance for larger models, but degrades performance for smaller models, evidenced by missed detections. The red dashed lines represent the result of model trained on real data. Scale bars represent 1 mm. Source data.

We also investigated the impact of synthetic background quality on downstream CV models (Extended Data Fig. 3). For MMDs with distinct features, such as stents, performance is largely unaffected by background quality, whereas for MMDs with ambiguous features, the effect is model-dependent: smaller models degrade with low-quality data, while larger models can utilize it as effective regularization. To tackle this issue, we adopt a two-phase quality control strategy during tissue generation (diffusion model selection and artefact minimization) and prioritize the utilization of large downstream models with its robustness to noise (‘Diffusion model training and inference’ in Methods). A classifier can be developed to automatically select backgrounds for CV model training as a future step.

**Extended Data Fig. 4:**
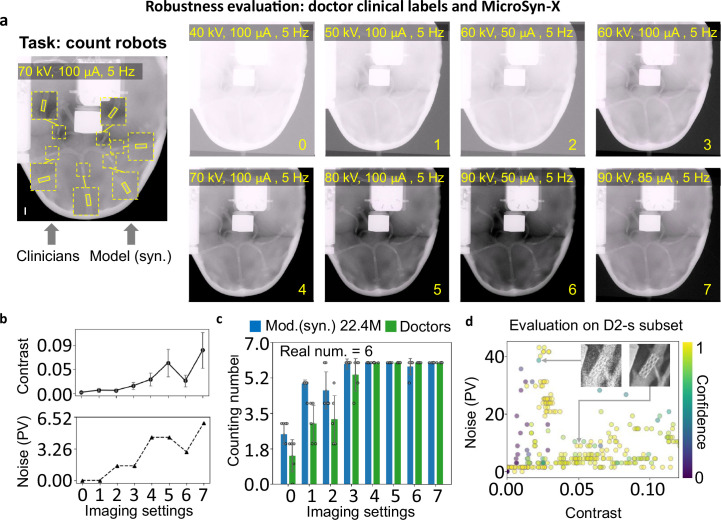
Robustness evaluation of MicroSyn-X.a. Evaluation task comparing clinical expert labels with MicroSyn-X. Videos of six static robots inside a skull model were recorded across varying imaging parameters for quantifying detectable robots. The numbers represent the indexes of imaging setting. **b**. The imaging effect of MMDs under different imaging settings. Error bars represent standard deviation across six different MMDs. **c**. Expert-label comparison. Model-derived robot counts and expert annotations are evaluated across eight imaging parameters. Error bars represent standard deviation across five clinicians and model detection confidences. **d**. Detection confidence distribution relative to contrast and noise levels. Effective detections require a segmentation mask IoU > 0.5 with ground truth. A subset of dataset D2-s was manually annotated, with defined contrast and noise computation regions defined. In all figures, data are presented as mean values $\pm$ standard deviation. Source data.

To evaluate the clinical relevance, we benchmarked its performance with experts in low-contrast and high-noise environments. Six soft MMDs were placed within a three-dimensional (3D) lumen phantom (Extended Data Fig. 4a,b) and imaged across varying X-ray voltages and currents (Supplementary Fig. 8). Both clinical experts and the CV model were tasked with counting visible MMDs: experts manually annotated images, while the model required segmentation with an IoU >0.5 for valid predictions. Quantitative analysis revealed that the model outputs matched expert consensus (Extended Data Fig. 4c). For soft MMDs in dataset D2-s, a subset was manually annotated (Supplementary Fig. 9 and ‘Computation of metrics’ in Methods). The CV model outputs aligned with manual identification (Extended Data Fig. 4d), reliably detecting MMDs when contrast exceeded 0.018, a threshold challenging for operators owing to signal degradation. These results validate the clinical applicability of MicroSyn-X.

### Fluoroscopy-guided robotic deployment

**Fig. 4:**
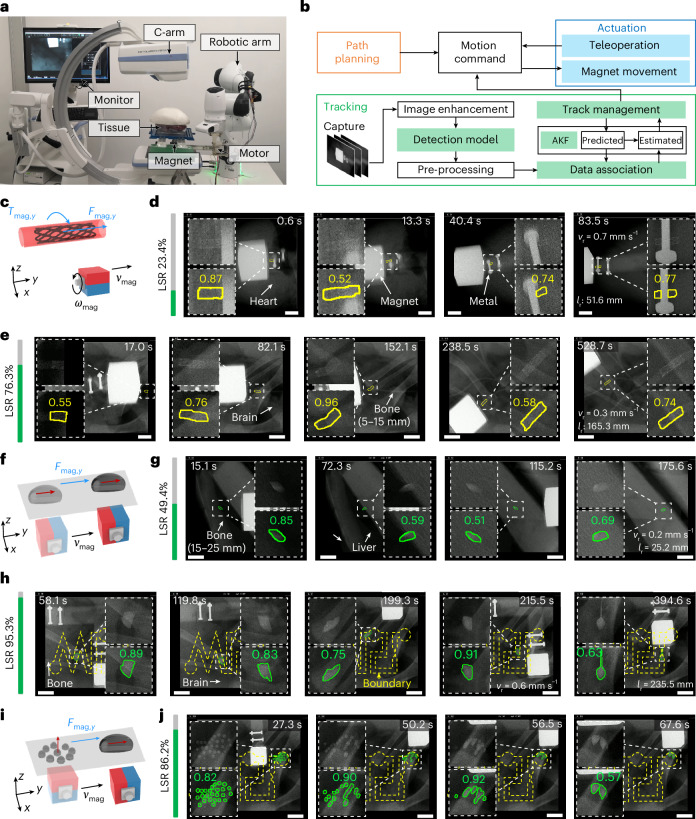
Robotic system integrated with MicroSyn-X-trained models. **a**, The X-ray-guided robotic actuation system. **b**, A schematic of the supervised autonomous robotic navigation system. **c**, The auction principle of the soft MMD utilizing magnetic torque and force. Using a rotating 30-mm N45 cubic magnet, the magnetic torque and force range from 2.0 μNm to 13.2 μNm and from 0.1 mN to 0.4 mN, respectively. Soft MMD dimensions were 1.5 mm in diameter and 5.0 mm in length. The axes *x*, *y* and *z* define the coordinate system. *F*_mag,y_ and *T*_mag,y_ denote the magnetic force and torque along the *y*-directon, respectively, while *v*_mag_ and *ω*_mag_ represent the magnet’s translational velocity and rotational speed. **d**, Real-time soft MMD tracking under high-occlusion, noisy and low-contrast imaging conditions. The localization success ratio (LSR), defined as the ratio of successfully localized frames to total video frames, is displayed alongside. The top row illustrates zoomed-in MMD views, while the bottom row shows segmentation results with confidence scores. **e**, Robotic navigation and real-time tracking within tortuous lumen networks under bone occlusion conditions. **f**, The magnetic gradient-driven translation of a ferrofluidic MMD. The red arrow represents the polarization direction of the magnetic field. Using a 20-mm N45 cubic magnet, the magnetic gradient along the desired motion direction ranges from 0.06 to 0.12 T m^−1^. The liquid MMD volume is 40–60 μl. **g**, Real-time liquid MMD tracking beneath bone structures up to 25 mm in thickness. **h**, Liquid MMD tracking in environments with abrupt spatial variations under bones in real time. The MMD morphology dynamically adapts to structural boundaries, such as the narrow-channel segment ‘I’ of the MPI configuration, enabling navigation through confined pathways. **i**, The liquid MMD separation and recombination via magnetic field modulation. **j**, The real-time tracking of liquid MMD swarms and recombination dynamics under bone occlusion. Scale bars represent 10 mm. The mean MMD translation speed and locomotion distance are denoted by ${v}_{{\rm{r}}}$ and ${l}_{{\rm{r}}}$, respectively.

**Extended Data Fig. 5:**
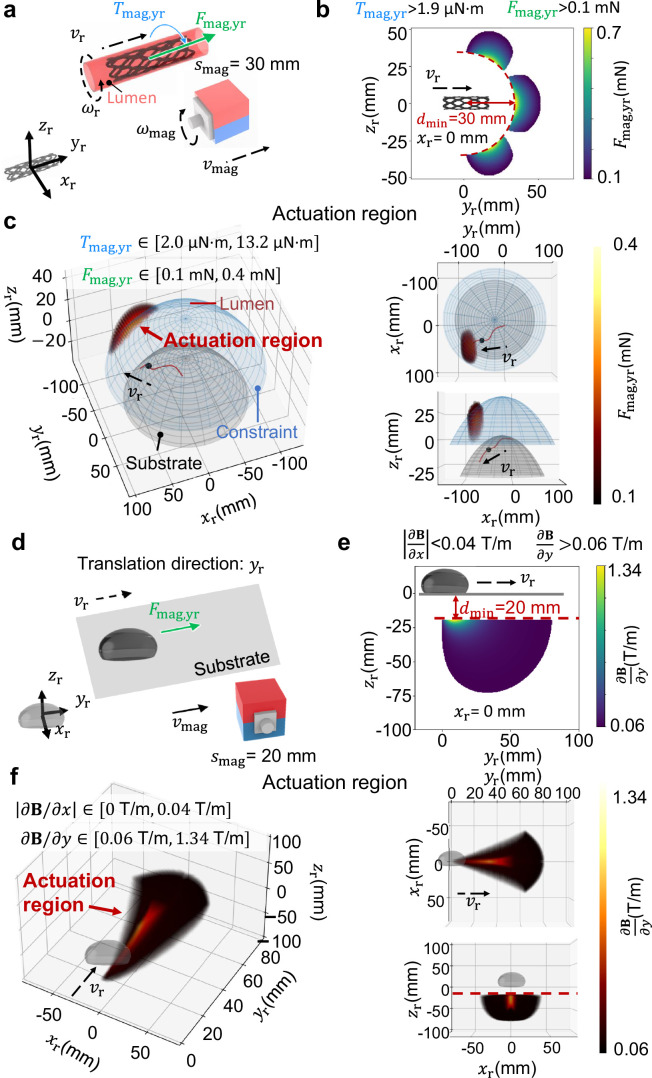
Actuation regions of soft and liquid MMDs. **a**. Actuation of the soft MMD inside a lumen, driven by magnetic torque and force. **b**. Actuation region of the soft MMD in the plane of ${x}_{{\rm{r}}}=0$. When the rotating permanent magnet (PM) is positioned within this region, the magnetic torque and force exceed the thresholds to enable MMD motion. **c**. Three-dimensional actuation region of the soft MMD, considering geometric constraints to prevent potential collisions. **d**. Actuation of the soft MMD on a substrate, where motion is achieved by utilizing the magnetic field gradient. **e**. Actuation region of the liquid MMD in the plane of ${x}_{{\rm{r}}}=0$. When the PM is located within this region, the magnetic field gradient along the desired direction exceeds the threshold to induce MMD movement. **f**. Three-dimensional actuation region of the liquid MMD. A larger PM can be employed to expand the actuation region. Source data.

Utilizing the CV model trained with MicroSyn-X, the telerobotic system translates vision-based localization into robotic deployment, integrating hardware and software to enable image-guided deployment of MMDs under X-ray imaging. In Fig. 4a, the system uses a robotic arm with ±1 mm precision, a permanent magnet (PM) mounted on a stepper motor and C-arm fluoroscopy for real-time navigation (‘Latency of the teleoperated robotic system’ in Methods). The software consists of four modules: planning, actuation, tracking and control (Fig. 4b). The planning module computes the MMD and PM paths^3^, utilizing preoperative data (computed tomography^102^ or rotational angiography^103^) and the MMD dynamic model (Extended Data Fig. 5). The actuation module drives PM translation and rotation to execute planned motions, while the tracking module localizes MMDs as feedback. The control module implements supervised autonomy: operators issue high-level commands (for example, ‘advance to the next waypoint’), while the system autonomously executes manipulation and localization. This hybrid scheme maintains operator oversight, as required in clinical workflows^8^, while enhancing precision and repeatability through automation.

**Extended Data Fig. 6:**
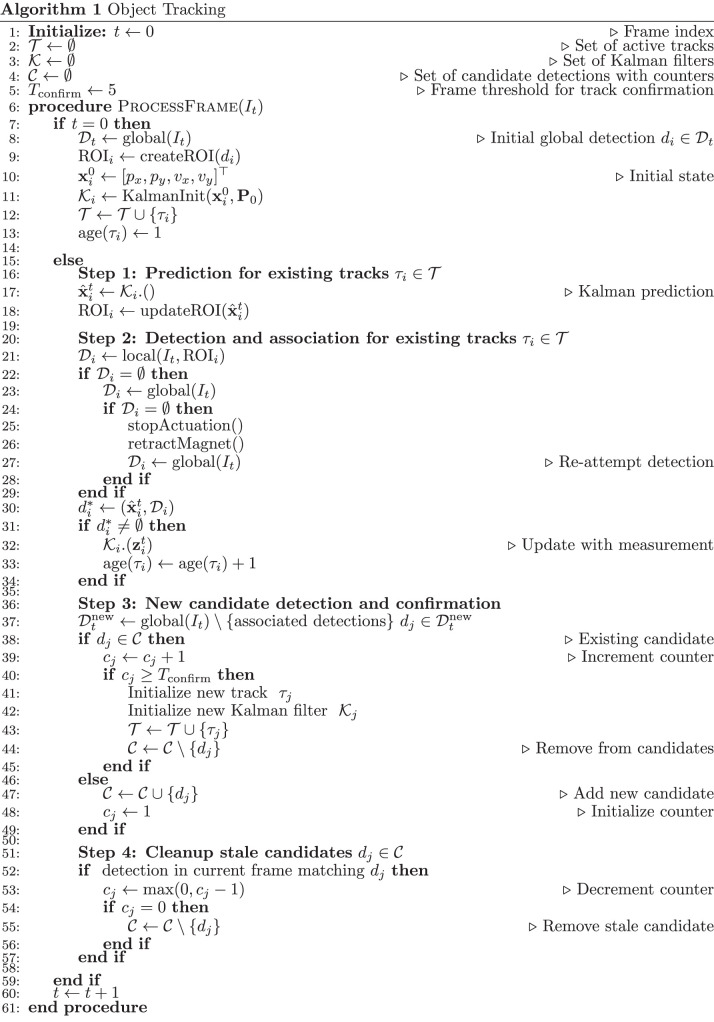
**Pseudocode of the object tracking algorithm**.

In detection-based MMD tracking, the CV model localizes the MMD in individual frames (Supplementary Fig. 2) and the tracking algorithm links these detections into trajectories. To handle the dynamic, low-contrast and noisy imaging environment with frequent occlusions, the system mitigates false positives, missed detections and abrupt appearance changes through several strategies. Each frame is preprocessed (for example, brightness/contrast adjustment and histogram equalization) to enhance MMD visibility^97^. Detection outputs are filtered by confidence scores, geometric consistency, spatial plausibility and temporal persistence, and the adaptive Kalman filter interpolates missing data during occlusions^104^ (Extended Data Fig. 6 and ’Measures for handling degradation in image quality’ in Methods).

The robotic system demonstrates robust deployment of MMDs in clinically relevant scenarios. For soft MMDs, a rotating PM generates magnetic torque and force for navigating complex anatomical pathways^3^ (Fig. 4c and Extended Data Fig. 5). Supplementary Movie 1 demonstrates reliable tracking across diverse tissue types despite varying tissue textures, imaging noise and partial occlusions. To validate tracking robustness under extreme conditions, stress tests were conducted in low-contrast, high-noise and severe occlusion environments. In Fig. 4d, the PM rotated at 1.3 Hz, inducing rapid occlusions and degraded visibility. In Fig. 4e,a soft MMD navigated contrast agent-filled lumens, traversing bifurcations and reversing direction under persistent bone-induced occlusions. Despite these adversities, the tracking algorithm maintained uninterrupted localization, demonstrating its capacity to handle non-detections and false positives (Supplementary Movie 2).

Ferrofluid-based liquid MMDs exhibit exceptional deformability, allowing adaptation to complex terrains but posing challenges for tracking. As shown in Fig. 4f and Extended Data Fig. 5, magnetic gradients generated by the PM drive droplet translation, while controlled magnetic field orientation induces shape deformation to navigate uneven or confined spaces. Supplementary Movie 3 and Fig. 4g demonstrate successful tracking as it traverses a 25-mm thick bone phantom, even at occluded boundaries. In Fig. 4h and Supplementary Movie 4, the MMD navigated an ‘MPI’-shaped structure with randomized bone occlusions (where MPI stands for Max Planck Institute), deforming substantially to pass through narrow channels while remaining tracked. The system also supports dynamic splitting and merging of ferrofluid droplets^91^ (Fig. 4i). A strong vertical magnetic field (from PM proximity) generates internal repulsive forces exceeding surface tension, splitting the droplet into smaller units^105^. Subsequent horizontal PM polarization initiates reassembly^105^. As shown in Fig. 4j and Supplementary Movie 4, swarm droplets were constantly tracked during the merging process under persistent bone and magnet occlusion. These results underscore the pipeline’s capability to track highly deformable objects in constrained, high-occlusion scenarios.

### Robotic deployment and tracking in ex vivo and in vivo tissues

To test the robotic deployment and tracking framework in realistic tissue environments, we conducted experiments in ex vivo and in vivo settings. A soft MMD was first deployed in a 3D curved porcine artery, then three MMDs were sequentially deployed beneath a skull model. In dynamic in vivo scenarios, a soft MMD navigated the rabbit femoral artery under physiological motion, while long-distance deployment in the rat abdominal aorta and iliac artery verified robust tracking despite severe bone occlusion and imaging degradation.

**Fig. 5:**
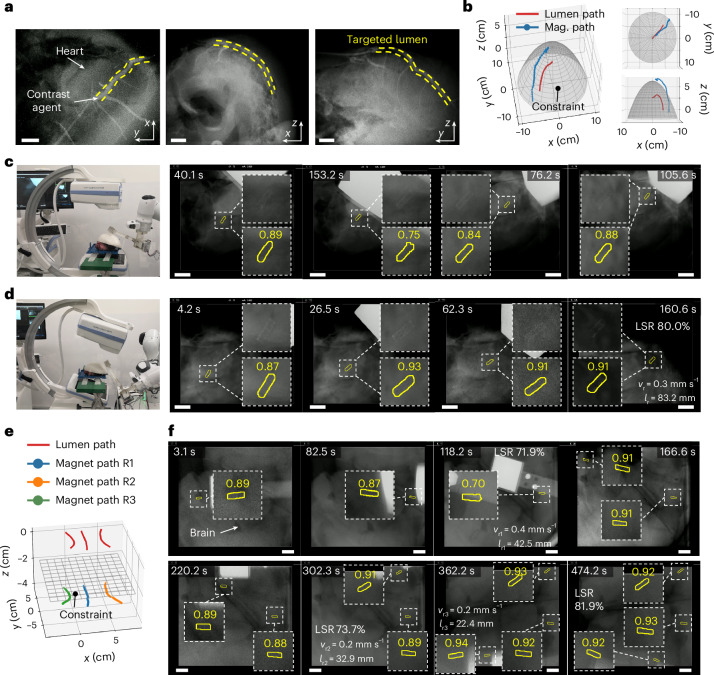
Robotic navigation in 3D ex vivo tissues and multi-MMD deployment. **a**, A 3D reconstruction of vascular pathways via intra-vascular contrast agent injection and multi-angle imaging. **b**, A reconstructed lumen path and planned magnet path for targeted robotic deployment. **c**., MMD deployment under low-contrast imaging conditions. Left: imaging-angle configuration. Top row insets: magnified MMD views. Bottom row insets: segmentation outputs with confidence scores. **d**, Real-time MMD tracking under imaging-angle misalignment. The segmentation neural network trained on fixed-angle MMD imagery demonstrates robust generalization to appearance variations induced by 3D rotational misalignment. Left: imaging-angle configuration. Top row insets: magnified MMD views. Bottom row insets: segmentation outputs with confidence scores. **e**, The path planning for multi-MMD deployment. **f**, Real-time multi-MMD deployment and tracking. Scale bars represent 10 mm. The mean MMD translation speed and locomotion distance are denoted by ${v}_{{\rm{r}}}$ and ${l}_{{\rm{r}}}$, respectively. Using a rotating 30-mm N45 cubic magnet, the magnetic torque and force range from 2.0 μNm to 13.2 μNm and from 0.1 mN to 0.4 mN, respectively. Soft MMD dimensions of 1.5 mm in diameter and 5.0 mm in length. Source data.

For robotic deployment in ex vivo tissues, the contrast agent was injected into the porcine heart artery, imaged from multiple angles and the 3D path was reconstructed by correlating the centreline of the contrast-enhanced regions (Fig. 5a). The path planning algorithm further computed PM trajectories optimized for magnetic actuation, considering the workspace constraints^3^ (Fig. 5b). The robotic system then executed user commands, guided by tracking results, to steer the PM along the planned path (Fig. 5c). To maximize visibility during deployment, the C-arm angle was adjusted to align with the MMD position. Despite low-contrast and noisy imaging conditions, the MMD was continuously tracked (Fig. 5d and Supplementary Movie 5). In Fig. 5e,f, three soft MMDs were sequentially deployed and tracked simultaneously in separate lumens, with all MMDs remaining tracked despite occlusions (Supplementary Movie 6).

A hybrid robotic deployment strategy is proposed for navigation in environments in vivo, integrating a customized mechanical device with fluoroscopic guidance and magnetic actuation. The system employs a suture with tailored flexibility and biocompatible materials to enable fluid-driven locomotion while ensuring fail-safe control during clinical interventions (Supplementary Fig. 10). A soft MMD (550 µm outer diameter) is delivered into the vasculature via an artery sheath, after which blood flow is harnessed for passive advancement under real-time fluoroscopic imaging. Directional control is achieved using a static magnet to guide the MMD to navigate through bifurcations to enter desired branches. In small blood vessels with insufficient haemodynamic force, the rotating PM generates magnetic torque and force to overcome resistance, enabling active navigation in low-flow environments. This dual-mode approach, combining physiological fluid dynamics with external magnetic actuation, enhances adaptability in complex anatomical settings.

**Fig. 6:**
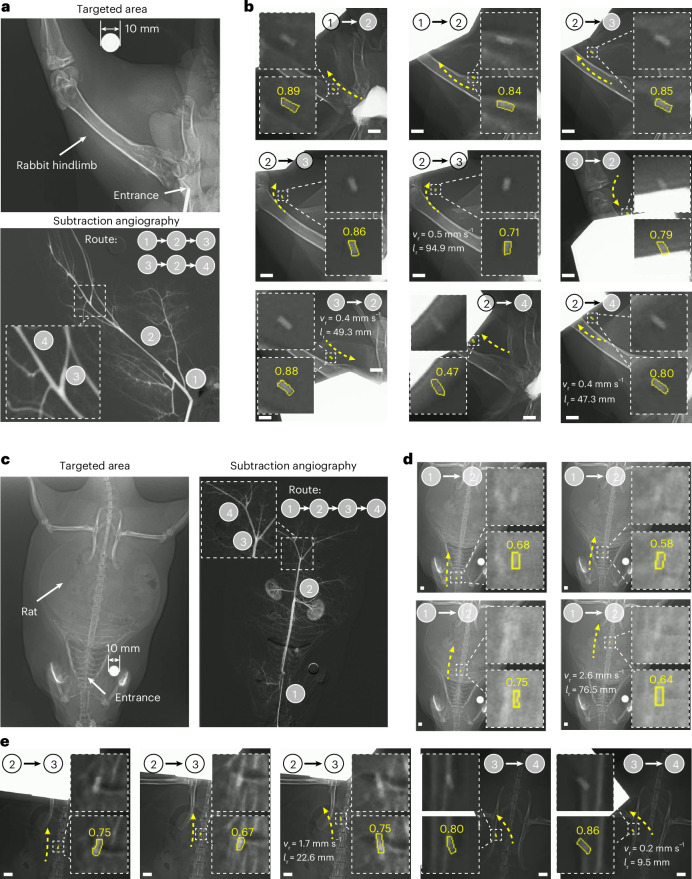
Robotic navigation in arterial environments in vivo. **a**, The targeted rabbit femoral arterial network with numbered waypoints during navigation. **b**, Navigation in dynamic in vivo conditions. Top row insets: magnified intra-arterial MMD views. Bottom row insets: segmentation outputs with confidence metrics. **c**, The targeted in vivo rat arterial region. **d**, The MMD deployment leveraging blood flow in the abdominal aorta. **e**, Magnetically actuated MMD navigation in the abdominal aorta and lilac artery. Scale bars represent 5 mm. The mean MMD translation speed and locomotion distance are denoted by ${v}_{{\rm{r}}}$ and ${l}_{{\rm{r}}}$, respectively. Using a rotating 60-mm N45 cubic magnet, the magnetic torque and force range from 0.46 μNm to 4.11 μNm and from 0.05 mN to 0.11 mN, respectively. Soft MMD dimensions of 0.55 mm in diameter and 1.80 mm in length.

The first in vivo demonstration involved a soft MMD navigating the rabbit femoral arterial network (Fig. 6a). Utilizing rotating PM actuation, the MMD traversed complex vascular structures (Fig. 6b and Supplementary Movie 7), where it entered two branches and executed bidirectional locomotion. In the second experiment, a rat model was used (Fig. 6c), where the MMD was delivered into the abdominal aorta and propelled by blood flow to waypoint 2 before entering a bifurcation. Despite a large imaging window, low imaging resolution and continuous spinal occlusion, the tracking algorithm achieved effective localization (Fig. 6d). Under combined magnetic guidance and fluid dynamics, the MMD entered the bifurcation area, after which the rotating PM actuated it to the distal target area. Detailed evaluation results are shown in Supplementary Fig. 11. Histology and biocompatibility analysis confirm the safety, biocompatibility and haemocompatibility^9^ (Supplementary Fig. 12 and ‘Histological examination’ in Methods). These results underscore the robustness of the tracking algorithm in clinically relevant scenarios, highlighting the potential of soft MMDs for minimally invasive vascular interventions. All locomotion data are summarized in Supplementary Tables 1 and 2.

## Discussion

Medical imaging-guided deployment of MMDs in physiological environments faces challenges such as detecting tiny, low-contrast objects in noisy, occluded scenes, limiting real-time tracking and precise control. Thus, we have developed a synthetic data generation pipeline (MicroSyn-X) to train CV models for robotic MMD navigation. Using diffusion-based synthesis, MicroSyn-X generates realistic X-ray scenes with automatic, pixel-accurate labels. Domain randomization broadens simulated physiological conditions, improving generalization to unseen clinical settings. The framework has been validated on soft and liquid MMDs in ex vivo and in vivo tissues, achieving performance comparable to clinical experts. Integrated into a robotic system, MicroSyn-X enables multi-robot navigation in 3D lumens and continuous tracking under bone occlusion and in vivo. In addition, we have released the open-sourced MMD dataset to foster reproducibility in medical robotics.

The proposed framework advances MMD localization and deployment under X-ray fluoroscopy by overcoming key limitations of existing methods. MicroSyn-X expands medical data synthesis to MMD-specific conditions, generating high-fidelity X-ray images that incorporate realistic noise, occlusion and low-contrast scenarios. It bridges the synthetic-to-real gap, demonstrating the feasibility of training models exclusively on synthetic data to perform robustly in clinical environments. With this framework, inexpensive high-quality data with large volume, expanded distribution and accurate labelling is obtained to train generalizable downstream models. Furthermore, the robotic system serves as a functional platform for translating these advancements into clinical applications. This work facilitates clinical translation of MMDs in minimally invasive procedures, targeted therapies and diagnostics.

The proposed system can be improved in multiple aspects. First, more advanced generative models and physics-based deformation models can be adopted to produce more realistic X-ray images that closely mimic real-world anatomical and device-specific features^106^. Moreover, integrating domain knowledge, such as biomechanical models of tissue deformation, could generate time-resolved datasets reflecting physiological motion^101,107,108^. Furthermore, advanced image fusion techniques could seamlessly embed MMDs into anatomical backgrounds with accurate 3D poses and textures^109^, while multimodal fusion with ultrasound or magnetic resonance imaging could address X-ray limitations in capturing microscopic biological interactions^110,111^. Second, other downstream CV models, such as transformer-based models^37^, can be utilized to enhance the tracking performance. Temporal models for video-based tracking could shift from frame-wise detection to continuous localization, improving efficiency in dynamic fluoroscopic sequences^112,113^, while extending MicroSyn-X to 3D segmentation could enable navigation in volumetric X-ray data^114^. Third, reinforcement learning could be adopted for autonomous MMD control^46^, along with digital twin interfaces to facilitate better visualization^115^. Last, more comprehensive in vivo validation is necessary to evaluate the system’s effectiveness in rare anatomical pathologies and long-term biocompatibility.

## Methods

### Hardware of the teleoperated robotic system

The six-degree-of-freedom (DOF) magnetic actuation platform comprises a cubic permanent magnet (N45, IMPLOTEX GmbH) driven by a NEMA 17 stepper motor (RS Components) and mounted on a 7-DOF robotic arm (Panda, Franka Emika GmbH). Visual feedback was provided by a C-arm fluoroscopy system (Fluoroscan InSight FD, Hologic GmbH). System control was split between a slave computer for robotic arm control and a host computer for fluoroscopic visualization, user input and command transmission. This configuration enables precise six-degree-of-freedom magnetic field control at the end-effector. During navigation, fluoroscopy settings were maintained above 50 kV and 50 µA, and MMD translation speed was limited to <3 mm s^−1^ to minimize motion blur (Extended Data Fig. 1).

Static MMD datasets were acquired using an X-ray cabinet system (XPERT 80, KUBTEC Scientific). A 20-mm cubic magnet (N45, IMPLOTEX GmbH) was actuated using two translational motorized stages (LTS300/M, Thorlabs Inc.) equipped with a stepper motor (535-0372, RS Components GmbH) and a servo motor (SKU 900-00360, Parallax Inc.). Sample height adjustments were performed using an additional LTS300/M translational stage. Together, these components constituted a 5-DOF robotic system^3^.

### Diffusion model training and inference

The diffusion model was trained to generate realistic X-ray tissue backgrounds conditioned on anatomical masks and textual prompts (Supplementary Fig. 3). The image number for each tissue category was less than 20 (Supplementary Fig. 13), and each image was automatically segmented into three channels representing tissue regions (${M}_{\mathrm{tissue}}$), metallic devices (${M}_{\mathrm{device}}$) and lumens with contrast agents (${M}_{\mathrm{lumen}}$), using thresholding-based methods optimized for each channel. To enhance dataset diversity and improve texture learning, geometric transformations and colour–space augmentations were applied during training. A programmatic prompt generation strategy was implemented to automate textual conditioning. A small, fixed vocabulary of anatomical terms (for example, ‘brain’, ‘skull’, ‘vessel’ and ‘lumen’) was curated once, and prompts were dynamically assembled through random combinations of these terms during data generation (for example, ‘porcine brain within the skull’ and ‘lumen inside heart’). This approach eliminated per-image manual input and maintained constant human effort regardless of dataset size. Future extensions may incorporate large language models to further enrich prompt diversity^116^.

A two-phase quality control strategy was applied during model training and inference. During training, candidate models were periodically evaluated using the SSIM to assess fidelity to real tissue images, Inception V3 feature distributions were visualized via PCA to confirm expanded but consistent domain coverage and qualitative screening was used to ensure realistic textures, illumination and contrast. During inference, generation parameters—including diffusion steps and classifier-free guidance scale (*ρ*)—were tuned to minimize artefacts such as blurriness, grid-like patterns, inconsistent illumination or overly smooth textures (Supplementary Fig. 14).

### Latency of the teleoperated robotic system

We quantified the latency of each processing step to assess the system’s real-time performance and stability during dynamic locomotion. The end-to-end process consists of three main stages: image acquisition, image processing and actuation command execution.

#### Image acquisition

X-ray imaging was performed using a C-arm system (Fluoroscan InSight FD, Hologic GmbH) operating at adaptive frame rates depending on the imaging mode. Under a continuous high-resolution mode, the frame rate ranged from 0 to 15 frames per second (f.p.s.), corresponding to a minimum interval of 66.7 ms per frame. Under a continuous standard-resolution mode, the system operated at 0–30 f.p.s. with a minimum frame interval of 33.3 ms, as specified in the manufacturer’s datasheet.

#### Image processing

Object localization was achieved using a dual-mode tracking algorithm (Supplementary Fig. 2 and Extended Data Fig. 6). In the local tracking mode—the primary operational mode focused on regions of interest (ROIs)—the average processing time was 21.6 ± 1.6 ms per object (model size of 22.4 M), measured from raw data input to result output. In the global re-initialization mode, used sparingly for comprehensive searches across the entire image, the latency was 333.8 ± 4.9 ms (model size of 22.4 M, 25 patches). All computations were performed on a workstation equipped with an NVIDIA RTX Titan GPU, Intel Xeon 5220 CPU at 2.2 GHz, and 64 GB RAM. The processing throughput is fully compatible with the X-ray acquisition rate of up to 30 f.p.s.

#### Actuation command

For clinical safety, the system incorporates a user-in-the-loop protocol, requiring operator approval before movement execution. After approval, the latency from command dispatch to robotic arm actuation was measured to be <112 ms. The robot’s locomotion speed remained below 1.5 mm s^−1^, and new commands were issued after the robot advanced approximately 1–2 mm. This latency is well within acceptable limits for stable dynamic locomotion.

### MMD data preparation and integration

The stent-structured soft MMDs were fabricated by moulding^9^, while the oil-based ferrofluids with a density of 1.43 g cm^−^^3^ and dynamic viscosities of 8 mPas were from Ferrotec Corporation (Supplementary Fig. 15). As shown in Supplementary Fig. 1, the soft MMDs were imaged with a clean background under X-ray cabinet imaging (XPERT 80, KUBTEC Scientific) with varying voltages and currents. Subsequently, the MMD regions in the resulting images were automatically segmented using the automated thresholding algorithm^97^. The largest MMD contour by area was extracted and rotated horizontally to standardize orientation, with blank regions cropped to isolate the soft MMD data. Ferrofluid images were synthesized using spline curve interpolation.

MMD integration was conducted as in the following steps. First, the targeted MMD pixel value was calculated. Given the original pixel value ${v}_{{\rm{b}}}\left(x,y\right)$ of the background at a target pixel location $\left(x{,}y\right)$ and predefined thresholds ${v}_{\min }$ (lowest value) and ${v}_{\max }$ (highest value), the target value ${v}_{{\rm{t}}}$ was computed by sampling a random contrast multiplier $\rho \in \left[{\rho }_{\mathrm{low}},{\rho }_{\mathrm{high}}\right]$. The target pixel value was calculated with ${v}_{{\rm{t}}}\left(x,y\right)=\,\rho \times {v}_{{\rm{b}}}\left(x,y\right)$, and ${v}_{{\rm{t}}}\left(x,y\right)$ is clipped between ${v}_{\min }$ and ${v}_{\max }$. Then, MMD selection and geometric transformation are performed. For each insertion position $\left(x,y\right)$ in the list of desired MMD locations: select or generate one MMD image instance $R$, scale $R$ so its height matches a predefined target height ($h$) and imposed random height perturbation and randomly rotate $R$ along with its mask $M$. Last, alpha blend and image composition were done with the blending coefficient $\alpha =\mathrm{sum}\left({v}_{{\rm{t}}}\left(x,y\right)-{v}_{{\rm{b}}}\left(x,y\right)\right)/\mathrm{sum}\left({v}_{{\rm{b}}}\left(x,y\right)-{v}_{{\rm{r}}}\left(x,y\right)\right)$, if $\left(x,y\right)\in M$ and ${v}_{{\rm{r}}}\left(x,y\right)$ is the pixel value of the MMD. The pixel value of the output image is computed with ${v}_{{\rm{b}}}\left(x,y\right)=\alpha \times {v}_{{\rm{b}}}\left(x,y\right)+\left(1-\alpha \right)\times {v}_{{\rm{r}}}\left(x,y\right)$, if $\left(x,y\right)\in M$. This process automatically seeds MMD shapes into fluoroscopic images with randomized contrast, size, and location, while preserving control over minimum contrast differences and masking boundaries.

### Measures for handling degradation in image quality

To ensure reliable tracking under dynamic and occasionally low-contrast imaging conditions, we implemented a multi-layered strategy that integrates software- and hardware-level controls. This framework mitigates failures arising from sudden drops in image quality, as systematically characterized in Extended Data Fig. 1, where low voltage or current, high frame rate and rapid MMD motion (>20 mm s^−1^) were identified as primary contributors to degraded fluoroscopic visibility.

#### Software strategies

The tracking algorithm incorporates a filtering module designed to reject false detections caused by poor image quality. Each detection is evaluated using multiple criteria, including: (1) a minimum confidence threshold from the computer vision model, (2) geometric consistency of the detected MMD (width, length and overall dimensions), (3) temporal continuity on the basis of the distance between the current and previous positions, (4) anatomical plausibility relative to the lumen centerline and (5) the recent historical localization success rate computed over the past ten frames. These filtering steps complement the preprocessing pipeline (for example, brightness/contrast adjustment and histogram equalization) and are integrated with the adaptive Kalman filter, which interpolates missing positions during occlusions. The complete algorithmic workflow and pseudocode are provided in Extended Data Fig. 6.

#### Hardware and protocol strategies

To minimize image degradation, fluoroscopy was operated above 50 kV and 50 µA (Extended Data Fig. 1). MMD translation speed was limited to <3 mm s^−1^ to reduce motion blur. If tracking was lost for extended periods, magnetic actuation was adjusted by reducing rotation frequency or repositioning the magnetic field to mitigate occlusion. During continuous acquisition, the fluoroscopy frame rate automatically adapted to object motion, switching to lower frame rates when needed to improve image quality.

#### Operator intervention

If an MMD remains undetected despite the above automated controls, the operator may manually adjust the C-arm angle to improve the imaging perspective, increase voltage and current for enhanced contrast or modify the fluoroscopic field of view. This manual adjustment pathway is also illustrated within the full tracking workflow in Extended Data Fig. 6.

The interactions between detection, filtering and trajectory reconstruction are summarized in Supplementary Fig. 2 and detailed in Extended Data Fig. 6.

### Mask generation with spline curves

The shapes of liquid MMDs and mask of tissue background were programmatically generated with the following steps (Supplementary Fig. 1). The first step was to generate $$n$$ approximately evenly distributed but randomly perturbed points on a circle of radius $$r$$ around a centre $$c=\left({c}_{x},{c}_{y}\right)$$, denoted by $${P}_{i}=\left({x}_{i},{y}_{i}\right)$$ for $$i={0,1},{\boldsymbol{\ldots}},n-1$$. Angular sector sampling was first done by uniformly sampling angles around the centre with $${\theta}_{i}\sim {\mathscr{U}}(i(2{{\pi}}/n),(i+1)2{{\pi }}/n)$$, after which the radial sampling was performed with $${r}_{i}\sim {\mathscr{U}}\left({l}_{\min },{l}_{\max }\right)$$, where $${l}_{\min },{l}_{\max }$$ are the lower and upper limits of the radius, respectively. After these two steps, each point was calculated with $${x}_{i}={c}_{x}+{r}_{i}\cos {\theta }_{i},{y}_{i}={c}_{y}+{r}_{i}\sin {\theta }_{i}$$, and all points were arrange into the array $${\bf{P}}={\left\{{\left(\right.x}_{i},{y}_{i}\right\}}_{i=0}^{n-1}$$. To produce a smooth, closed curve through $$P$$, the first point was appended to the end and then fit a periodic B-spline with $${\bf{P}}^{\prime}=\left[\left({x}_{0},{y}_{0}\right),\ldots ,\left({x}_{n-1},{y}_{n-1}\right),\left({x}_{0},{y}_{0}\right)\right]$$. Subsequently, a periodic, smoothing-free ($$s=0$$) B-spline was computed and represented as $${\bf{C}}\left(u\right)=\left(X\left(u\right),Y\left(u\right)\right),u\in \left[\mathrm{0,1}\right],$$ such that $$C\left({u}_{j}\right)={P}_{j}^{{\prime} }$$ for a knot vector $${u}_{j}$$ of length $$n+1$$. With the computed B-spline curve^117^, $${N}_{{\rm{interp}}}$$ interpolated curve points were uniformly re-sampled as $$({x}_{k}^{\left(\mathrm{new}\right)},{y}_{k}^{\left(\mathrm{new}\right)})=(X({u}_{k}^{\left(\mathrm{new}\right)}),Y({u}_{k}^{\left(\mathrm{new}\right)}))$$, with $${u}_{k}^{({\mathrm{new}})}=k /(N_{\mathrm{interp}}-1)$$, and $$k=0,\ldots ,$$$${N}_{\mathrm{interp}}-1$$. These points were arranged as the final contour as $${{\bf{C}}}_{\mathrm{interp}}={\{({x}_{k}^{\left({\mathrm{new}}\right)},{y}_{k}^{\left({\mathrm{new}}\right)})\}}_{k=0}^{{N}_{\mathrm{interp}}-1}$$.

### Dataset preparation

Synthetic data were automatically generated and used to train a model referred to as model (syn.). Real MMD data were divided into soft and liquid types, each imaged under static and locomotion conditions. Static imaging placed MMDs in phantoms or biological tissues using an X-ray cabinet system (XPERT 80, KUBTEC Scientific). Locomotion imaging recorded videos with a C-arm system (Fluoroscan InSight FD, Hologic GmbH) mounted on a robotic arm (Panda, Franka Emika GmbH). Video frames were analyzed with model (syn.) and manually checked: outputs matching expert identification were adopted as labels, while discrepancies were manually annotated if the MMD was identifiable. Labels followed a one-text-file-per-image format, with each row indicating a single object’s class index and polygonal contour coordinates: class index, *x*_1_, *y*_1_, *x*_2_, *y*_2,_…, *x*_*n*_, *y*_*n*_.

For evaluating the model performance, ROIs that centred on the MMD or excluded the MMD were extracted. The locomotion dataset was categorized according to tissue type. For soft MMDs, tissue categories included porcine brain with embedded bones, porcine brain alone, heart, liver, stomach, heart 3D vessels, in vivo rat models and in vivo rabbit models. For liquid MMDs, tissue types encompassed porcine brain (with and without embedded bones), heart, liver and stomach, as well as scenarios involving MMD swarms under bone occlusion. The datasets and model weights (diffusion model and instance segmentation model) are available at ref. ^118^. Custom Python code was used for labelling and analyzing the data leveraging LABELME (5.5.0), MATPLOTLIB (3.7.3), NUMPY (1.24.4), SCIPY (1.10.1), PYTORCH (1.11.0) and PANDAS (1.4.4) packages.

### CV model training and inference

When the MMD occupied a small region within a large field of view, ROIs centred on the MMD were extracted for segmentation training. ROIs were split into training and validation sets at ratios of 20:1 for synthetic data and 10:1 for real data. Data augmentation included geometric transformations (scaling and translation of 0.5, rotation of ≤90° and shear of 30°), colour augmentation in the hue, saturation, value (HSV) space (brightness factor of 0.8) and robustness-enhancing operations such as perspective distortion (0.0002), vertical flipping (50%), random erasing (20%) and copy–paste augmentation (5%). Four model variants (2.8M, 10.1M, 22.4M and 27.6M parameters) were trained for 80 epochs with a batch size of 20 using a cosine learning rate scheduler (with an initial rate of 0.01 and a final rate of 0.0001).

For model inference, we implemented a dynamic dual-mode localization framework that alternates between processing the entire frame and focused patches to optimize both accuracy and speed. The overall workflow is illustrated in Supplementary Fig. 2 and Extended Data Fig. 6. In the ‘global search mode’, the frame is subdivided into overlapping patches for processing. This mode is used sparingly—for initialization or recovery for lost MMDs—to ensure comprehensive scene coverage. The ‘local tracking mode’ serves as the primary operational mode for real-time tracking. Once the MMD’s position is identified, the model processes only a cropped ROI around the last known position, rather than the entire frame. This hybrid strategy enables real-time performance by combining the speed of the local tracking mode with the robustness of the global search mode.

### Computation of metrics

For classification, each ROI is assigned to one of two classes: MMD or non-MMD. The maximum object detection confidence score within the ROI was used as the predicted probability of an MMD being present in the image. AP was used to evaluate the performance of binary or multi-class classification models by summarizing the precision-recall curve. AP calculates the weighted mean of precision values achieved at each confidence threshold, with weights determined by the change in recall between thresholds. Specifically, the score is derived using the formula $\mathrm{AP}={\sum }_{n}({r}_{n}-{r}_{n-1}))\cdot {p}_{n}$, where ${r}_{n}$ and ${r}_{n-1}$ represent consecutive recall values, and ${p}_{n}$ is the precision at threshold $n$.

Detection and segmentation performance were evaluated using mAP50(B) and mAP50(M), which measure mean AP for bounding boxes and masks, respectively, at an IoU threshold of 0.5. IoU is defined as the overlap between predicted and ground-truth regions divided by their union, with predictions considered correct when IoU was >0.5. More stringent metrics, mAP50:95(B) and mAP50:95(M), average mAP over IoU thresholds from 0.5 to 0.95 in 0.05 increments, thereby assessing robustness and spatial precision of both bounding boxes and segmentation masks.

Contrast and noise were calculated using region-based analysis, where three regions are manually defined: the object region ($${M}_{\mathrm{obj}}$$), background region ($${M}_{\mathrm{back}}$$) and noise region ($${M}_{\mathrm{noise}}$$). Contrast was determined using the Michelson contrast formula: $$\mathrm{contrast}$$$$=(\mathrm{mean}\left(v\left({x}_{\mathrm{obj}},{y}_{\mathrm{obj}}\right)\right)$$$$-\mathrm{mean}\left(v\left({x}_{\mathrm{back}},{y}_{\mathrm{back}}\right)\right))$$$$/(\mathrm{mean}\left(v\left({x}_{\mathrm{obj}},{y}_{\mathrm{obj}}\right)\right)+$$$$\mathrm{mean}\left(v\left({x}_{\mathrm{back}},{y}_{\mathrm{back}}\right)\right))$$, if $$\left({x}_{\mathrm{obj}},{y}_{\mathrm{obj}}\right)\in {M}_{\mathrm{obj}}$$ and $$\left({x}_{\mathrm{back}},{y}_{\mathrm{back}}\right)\in {M}_{\mathrm{back}}$$. For noise estimation, the median absolute deviation of pixel intensities in $${M}_{\mathrm{noise}}$$ was calculated as the median of absolute deviations from the median intensity, then scaled by 1.4826 to approximate the standard deviation of Gaussian noise.

### Ex vivo tissue phantom preparation

Organs were obtained as animal by-products (registration number DE 08 111 1008 21) under permits issued by the Stuttgart state authorities for food control, consumer protection and veterinary services. In compliance with permit requirements, biomaterial use was documented and all samples were pressure-sterilized after experiments. Coronary arteries for locomotion and mechanical testing were isolated from fresh porcine hearts within 48 h post-slaughter, stored at 4 °C, and sourced from Slaughterhouse Ulm (Germany) and Gourmet Compagnie GmbH (Germany). Before testing, tissues were rinsed with phosphate-buffered saline. For ex vivo experiments, phosphate-buffered saline (pH 7.4, Gibco, Thermo Fisher Scientific) was perfused through the arteries at 10–12 ml min^−1^, and angiographic imaging was performed using Iomeron 400 contrast agent (Bracco UK Limited).

Agarose gel samples with internal lumens were fabricated using 3D-printed positive moulds (Form 3, Formlabs Inc.). Agarose powder (A9539, Sigma-Aldrich) was dissolved in deionized water at 90 °C, boiled for 5 min, poured into Petri dishes containing the moulds, and cooled at room temperature (~24 °C) for 30 min before mould removal. The resulting agarose lumens were embedded in or placed beneath tissue samples to assess X-ray imaging performance of medical devices^3^.

### Setup for in vivo animal testing

This animal study was approved by the Committee on Institutional Animal Care and Use Committee of Hong Kong Huateng Biotechnology Co., Ltd. (IACUC number B202502-25) and the Institutional Animal Research Ethics Sub-Committee of City University of Hong Kong (AN-STA-00001025). New Zealand White rabbits (*Oryctolagus cuniculus*), outbred albino stock (genetic background: outbred), aged 4 months and weighing 2.5–3.0 kg at the time of experimentation, were obtained from Guangzhou Xindongxinhua Experimental Animal Breeding Farm (Guangzhou, China). Sprague Dawley rats (*Rattus norvegicus*), outbred stock (genetic background: outbred), aged 4 months and weighing approximately 700 g at the time of experimentation, were obtained from Zhuhai Bestone Biotechnology Co., Ltd. (Zhuhai, China). Both species are standard, commercially available outbred laboratory animals with no genetic modifications. All procedures were performed under anaesthesia to ensure animal welfare. The MMD device was introduced into the femoral artery of rabbits and the aorta of rats via a 4-Fr sheath (Glidesheath, Terumo), following a small incision. Real-time deployment was monitored using X-ray fluoroscopy (DSA, CGO-2100, Wandong Co. Ltd.). Upon reaching the targeted vascular region, the MMD was retrieved through magnetic actuation or by wire traction. Finally, the sheath was removed, and the surgical wound was sutured following standard protocols.

To ensure haemocompatibility and biocompatibility, the MMD surface was coated with a 1 µm layer of parylene C (SCS Labcoter 2, Specialty Coating Systems). Parylene C is Food and Drug Administration-approved for blood-contacting medical devices and has a long history of use in stents, guidewires and catheters^119–121^. Our previous work demonstrated that parylene C-coated polydimethylsiloxane films tolerate repeated large-deformation bending without delamination^9^, supporting short-term mechanical and chemical stability. Long-term stability under chronic physiological conditions, however, requires further investigation.

**Histological examination**

The MMD was injected into the femoral artery of the rabbit and rotated under the rotating magnetic field. The part of the artery contacting the MMD was cut out for histological examinations. Histological processing, performed by the Guangzhou Huitong Medical Code Pathology Diagnostic Center, included paraffin embedding, sectioning, hematoxylin and eosin staining, Masson’s trichrome staining, white light microscopy and qualitative analysis. The histological images (Supplementary Fig. 12) showed that the vascular lumen was open with no evidence of obstruction or thrombus formation. Endothelial cells of the vessel wall were arranged regularly, with no signs of hyperplasia or detachment. The internal elastic lamina was intact, with no signs of loss or rupture. The tunica media was composed of circumferentially arranged smooth muscle cells, showing no damage. The tunica adventitia consisted of loose connective tissue and appeared undamaged. Masson’s trichrome staining revealed no visible fibrous tissue proliferation in the intima, media or adventitia of the vessel wall. Furthermore, detailed examinations of biocompatibility and haemocompatibility were done in our previous studies^9^.

### Reporting summary

Further information on research design is available in the Nature Portfolio Reporting Summary linked to this article.

## Supplementary information

Supplementary Information Supplementary Figs. 1–15, Tables 1 and 2 and Notes for Supplementary Movies 1–8

Reporting Summary

Peer Review file

Supplementary Video1 Supplementary Movie 1. Soft MMD tracking in diverse ex vivo tissue models. This video demonstrates the navigation and tracking performance of stent-structured MMDs across various ex vivo biological tissues. The tracking capability was validated under challenging conditions, including bone with thicknesses up to 25 mm, as well as porcine heart, liver, stomach and brain tissues. Despite the diverse tissue textures, imaging noise, and partial occlusions, the algorithm maintained robust and accurate tracking throughout the experiments.

Supplementary Video2 Supplementary Movie 2. Soft MMD tracking in challenging imaging scenes. This video illustrates the navigation and tracking performance of stent-structured MMDs within a skull phantom featuring randomly placed bones to simulate dense anatomical obstructions. A soft MMD navigated through a contrast agent-filled lumen, successfully traversing bifurcations and reversing direction despite persistent occlusions caused by bone structures. In addition, the robustness of the tracking system was validated under frequent mechanical occlusions and degraded imaging quality.

Supplementary Video3 Supplementary Movie 3. Liquid MMD tracking in diverse ex vivo tissue models. This video showcases the navigation and tracking performance of ferrofluid-based MMDs across a range of ex vivo biological tissues. The tracking system was validated under challenging conditions, including bone with thicknesses up to 25 mm, as well as porcine heart, liver, stomach and brain tissues. Despite variations in tissue texture, imaging noise, partial occlusions and changes in MMD morphology, the MMDs were robustly tracked throughout the experiments.

Supplementary Video4 Supplementary Movie 4. Liquid MMD tracking in challenging imaging scenes. This video presents the navigation and tracking of a ferrofluid-based MMD within an MPI-shaped structure containing randomized bone occlusions. The MMD underwent substantial deformation to traverse narrow channels while remaining continuously tracked. In addition, dynamic behaviours such as the splitting and merging of swarm-like ferrofluid formations were tracked throughout the merging process, despite persistent occlusions from bone structures and external magnets.

Supplementary Video5 Supplementary Movie 5. Soft MMD deployment in 3D porcine arteries. This video demonstrates the deployment of a stent-structured MMD within a 3D-shaped ex vivo blood vessel, visualized using contrast-enhanced C-arm imaging from three orthogonal angles. A 3D vascular path was reconstructed from the contrast agent-filled lumen, providing an anatomical roadmap for navigation. Subsequently, a planning algorithm computed magnet trajectories for magnetic actuation within spatial constraints. The MMD followed user commands along the planned route, guided by real-time tracking, and the C-arm angle was adjusted to maintain visibility. Despite imaging noise, low contrast, partial occlusions and MMD 3D rotation, the MMD is continuously and accurately tracked.

Supplementary Video6 Supplementary Movie 6. Multi-MMD deployment in the ex vivo tissue model. This video demonstrates the system scalability through multi-MMD validation. Three soft MMDs were sequentially deployed and simultaneously tracked in separate lumens beneath a porcine brain and skull model, with robust tracking maintained despite occlusions.

Supplementary Video7 Supplementary Movie 7. Soft MMD deployment in the rabbit femoral arterial network in vivo. This video presents an in vivo demonstration of a soft MMD navigating the rabbit femoral arterial network, successfully traversing four predefined waypoints and multiple branches. Utilizing rotating magnetic actuation, the MMD entered two branches and performed bidirectional locomotion.

Supplementary Video8 Supplementary Movie 8. Soft MMD deployment in rat arterial regions in vivo. This video demonstrates a rat model in which the MMD was delivered into the abdominal aorta and carried by blood flow to a predefined waypoint before entering a bifurcation. Despite a large imaging window, low resolution and continuous spinal occlusion, the tracking algorithm maintained effective localization. Leveraging combined magnetic guidance and fluid dynamics, the MMD successfully navigated the bifurcation, after which a rotating permanent magnet actuated it towards the distal target area.

Supplementary Data Supplementary Data Tables 1–4, including the data for Supplementary Figs. 5–7 and 11.

## Source data

Source Data Fig. 3 Statistical source data.

Source Data Fig. 5 Statistical source data.

Source Data Extended Data Fig. 1 Statistical source data.

Source Data Extended Data Fig. 2 Statistical source data.

Source Data Extended Data Fig. 3 Statistical source data.

Source Data Extended Data Fig. 4 Statistical source data.

Source Data Extended Data Fig. 5 Statistical source data.

## Figures and Tables

**Extended Data Table 1:**
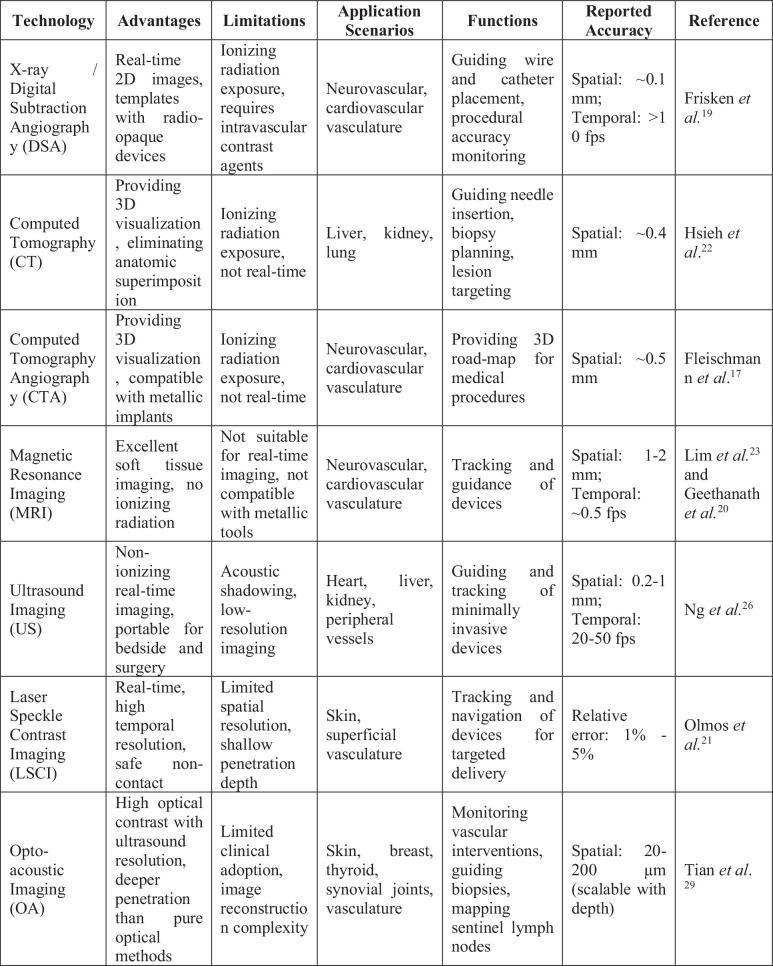
External sensing modalities for in vivo MMDs

**Extended Data Table 2:**
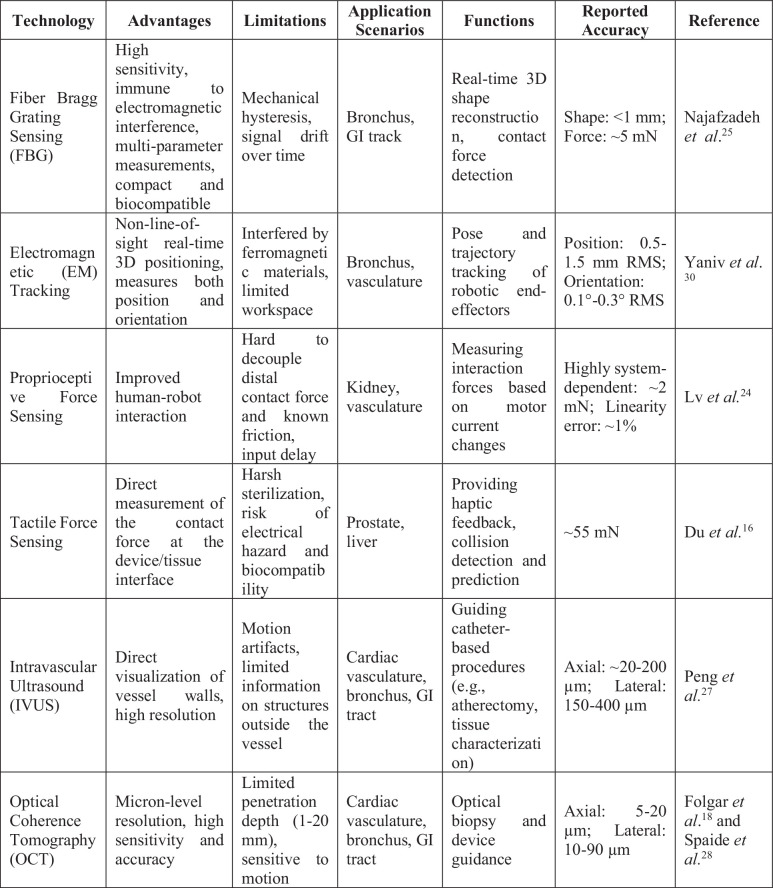
Intraluminal sensing modalities for in vivo MMDs

## Data Availability

The datasets used in this study—including the synthetic MMD data, real MMD data, real MMD locomotion data and other data referenced in the Methods section—are available at https://huggingface.co/datasets/luoyeguigenno1/X-ray-Miniature-Medical-Device (ref. ^118^). The repository also contains the trained model weights for the diffusion and instance segmentation models. Source data are provided with this paper.

## Code availability

The source code supporting this study is available at https://huggingface.co/datasets/luoyeguigenno1/X-ray-Miniature-Medical-Device (ref. ^118^), under the CC BY-NC 4.0 license.

## References

[1] Sitti, M. *Mobile Microrobotics* (MIT Press, 2017).

[2] Hu, W., Lum, G. Z., Mastrangeli, M. & Sitti, M. Small-scale soft-bodied robot with multimodal locomotion. *Nature* **554**, 81–85 (2018). 29364873 10.1038/nature25443

[3] Wang, C. et al. Heterogeneous multiple soft millirobots in three-dimensional lumens. *Sci. Adv.* **10**, eadq1951 (2024). 39504364 10.1126/sciadv.adq1951 PMC11540014

[4] Li, S. et al. Bioresorbable, wireless, passive sensors for continuous pH measurements and early detection of gastric leakage. *Sci. Adv.* **10**, eadj0268 (2024). 38640247 10.1126/sciadv.adj0268 PMC11029800

[5] Kwon, K. et al. A battery-less wireless implant for the continuous monitoring of vascular pressure, flow rate and temperature. *Nat. Biomed. Eng.* **7**, 1215–1228 (2023). 37037964 10.1038/s41551-023-01022-4

[6] Xu, C., Solomon, S. A. & Gao, W. Artificial intelligence-powered electronic skin. *Nat. Mach. Intell.* **5**, 1344–1355 (2023). 38370145 10.1038/s42256-023-00760-z PMC10868719

[7] Sitti, M. Miniature soft robots—road to the clinic. *Nat. Rev. Mater.* **3**, 74–75 (2018).

[8] Wang, T., Wu, Y., Yildiz, E., Kanyas, S. & Sitti, M. Clinical translation of wireless soft robotic medical devices. *Nat. Rev. Bioeng.* **2**, 470–485 (2024).

[9] Wang, T. et al. Adaptive wireless millirobotic locomotion into distal vasculature. *Nat. Commun.* **13**, 4465 (2022). 35915075 10.1038/s41467-022-32059-9 PMC9343456

[10] Ren, Z. et al. Soft-bodied adaptive multimodal locomotion strategies in fluid-filled confined spaces. *Sci. Adv.* **7**, eabh2022 (2021). 34193416 10.1126/sciadv.abh2022 PMC8245043

[11] Wu, Y., Dong, X., Kim, J. -k, Wang, C. & Sitti, M. Wireless soft millirobots for climbing three-dimensional surfaces in confined spaces. *Sci. Adv.* **8**, eabn3431 (2022). 35622917 10.1126/sciadv.abn3431 PMC9140972

[12] Wang, C., Wu, Y., Dong, X., Armacki, M. & Sitti, M. In situ sensing physiological properties of biological tissues using wireless miniature soft robots. *Sci. Adv.* **9**, eadg3988 (2023). 37285426 10.1126/sciadv.adg3988 PMC7614673

[13] Han, J. et al. Actuation-enhanced multifunctional sensing and information recognition by magnetic artificial cilia arrays. *Proc. Natl Acad. Sci. USA* **120**, e2308301120 (2023). 37792517 10.1073/pnas.2308301120 PMC10589697

[14] Yan, Y. et al. Magnetically assisted soft milli-tools for occluded lumen morphology detection. *Sci. Adv.* **9**, eadi3979 (2023). 37585531 10.1126/sciadv.adi3979 PMC10431716

[15] Dupont, P. E. et al. A decade retrospective of medical robotics research from 2010 to 2020. *Sci. Robot.* **6**, eabi8017 (2021). 34757801 10.1126/scirobotics.abi8017 PMC8890492

[16] Du, L. et al. An implantable, wireless, battery-free system for tactile pressure sensing. *Microsyst. Nanoeng.* **9**, 130–142 (2023). 37829157 10.1038/s41378-023-00602-3 PMC10564885

[17] Fleischmann, D., Chin, A. S., Molvin, L., Wang, J. & Hallett, R. Computed tomography angiography: a review and technical update. *Radiol. Clin.* **54**, 1–12 (2016). 10.1016/j.rcl.2015.09.002 26654388

[18] Folgar, F. A., Yuan, E. L., Farsiu, S. & Toth, C. A. Lateral and axial measurement differences between spectral-domain optical coherence tomography systems. *J. Biomed. Opt.* **19**, 016014 (2014). 24441877 10.1117/1.JBO.19.1.016014 PMC3894429

[19] Frisken, S., Haouchine, N., Du, R. & Golby, A. J. Using temporal and structural data to reconstruct 3D cerebral vasculature from a pair of 2D digital subtraction angiography sequences. *Comput. Med. Imaging Graph.* **99**, 102076 (2022). 35636377 10.1016/j.compmedimag.2022.102076 PMC10801782

[20] Geethanath, S. & Vaughan, J. T. Jr Accessible magnetic resonance imaging: a review. *J. Magn. Reson. Imaging* **49**, e65–e77 (2019). 30637891 10.1002/jmri.26638

[21] González Olmos, A., Zilpelwar, S., Sunil, S., Boas, D. A. & Postnov, D. D. Optimizing the precision of laser speckle contrast imaging. *Sci. Rep.* **13**, 17970 (2023). 37864006 10.1038/s41598-023-45303-z PMC10589309

[22] Hsieh, J. & Flohr, T. Computed tomography recent history and future perspectives. *J. Med. Imaging* **8**, 052109 (2021). 10.1117/1.JMI.8.5.052109 PMC8356941 34395720

[23] Lim, W. H., Park, J. S., Park, J. & Choi, S. H. Assessing the reproducibility of high temporal and spatial resolution dynamic contrast-enhanced magnetic resonance imaging in patients with gliomas. *Sci. Rep.* **11**, 23217 (2021). 34853347 10.1038/s41598-021-02450-5 PMC8636480

[24] Lv, C., Wang, S. & Shi, C. A high-precision and miniature fiber Bragg grating-based force sensor for tissue palpation during minimally invasive surgery. *Ann. Biomed. Eng.* **48**, 669–681 (2020). 31686311 10.1007/s10439-019-02388-w

[25] Najafzadeh, A. et al. Application of fibre Bragg grating sensors in strain monitoring and fracture recovery of human femur bone. *Bioengineering* **7**, 98–108 (2020). 32825200 10.3390/bioengineering7030098 PMC7552668

[26] Ng, A. & Swanevelder, J. Resolution in ultrasound imaging. *Contin. Educ. Anaesth. Crit. Care Pain* **11**, 186–192 (2011).

[27] Peng, C., Wu, H., Kim, S., Dai, X. & Jiang, X. Recent advances in transducers for intravascular ultrasound (IVUS) imaging. *Sensors* **21**, 3540–3555 (2021). 34069613 10.3390/s21103540 PMC8160965

[28] Spaide, R. F. et al. Lateral resolution of a commercial optical coherence tomography instrument. *Trans. Vis. Sci. Technol.* **11**, 28–28 (2022). 10.1167/tvst.11.1.28 PMC8787587 35044444

[29] Tian, C. et al. Impact of system factors on the performance of photoacoustic tomography scanners. *Phys. Rev. Appl.* **13**, 014001 (2020).

[30] Yaniv, Z., Wilson, E., Lindisch, D. & Cleary, K. Electromagnetic tracking in the clinical environment. *Med. Phys.* **36**, 876–892 (2009). 19378748 10.1118/1.3075829 PMC2673677

[31] Wang, C., Wang, T. & Sitti, M. Synthetic data-assisted miniature medical robot navigation via ultrasound imaging. *IEEE/ASME Trans. Mechatron.* **30**, 7717–7727 (2025).

[32] Zhang, Q. et al. Addressing the “world map problem” for submillimeter medical robots requires advancing local sensing beyond external imaging dependence. *Device* **3**, 100839 (2025).

[33] Martz, H. E., Logan, C. M., Schneberk, D. J. & Shull, P. J. *X-ray Imaging: Fundamentals, Industrial Techniques and Applications* (CRC Press, 2016).

[34] Seeram, E. *X-Ray**Imaging Systems for Biomedical Engineering Technology: an Essential Guide* (Springer, 2023).

[35] Razzak, M. I., Naz, S. & Zaib, A. in *Classification in BioApps: Automation of Decision Making* Vol. 26 (eds Dey, N. et al.) 323–350 (Springer, 2018).

[36] Shen, D., Wu, G. & Suk, H.-I. Deep learning in medical image analysis. *Annu. Rev. Biomed. Eng.* **19**, 221–248 (2017). 28301734 10.1146/annurev-bioeng-071516-044442 PMC5479722

[37] Min, Z., Lai, J. & Ren, H. Innovating robot-assisted surgery through large vision models. *Nat. Rev. Electr. Eng.* **2**, 1–14 (2025).

[38] Suzuki, K. Overview of deep learning in medical imaging. *Radiol. Phys. Technol.* **10**, 257–273 (2017). 28689314 10.1007/s12194-017-0406-5

[39] Zhou, S. K., Greenspan, H. & Shen, D. *Deep Learning for Medical Image Analysis* (Academic Press, 2023).

[40] Yang, L. et al. Autonomous environment-adaptive microrobot swarm navigation enabled by deep learning-based real-time distribution planning. *Nat. Mach. Intell.* **4**, 480–493 (2022).

[41] Schmidgall, S., Kim, J. W., Kuntz, A., Ghazi, A. E. & Krieger, A. General-purpose foundation models for increased autonomy in robot-assisted surgery. *Nat. Mach. Intell.* **6**, 1–9 (2024).

[42] Kim, Y. et al. Telerobotic neurovascular interventions with magnetic manipulation. *Sci. Robot.* **7**, eabg9907 (2022). 35417201 10.1126/scirobotics.abg9907 PMC9254892

[43] Han, Y. et al. Multi-animal 3D social pose estimation, identification and behaviour embedding with a few-shot learning framework. *Nat. Mach. Intell.* **6**, 48–61 (2024).

[44] Bello, G. A. et al. Deep-learning cardiac motion analysis for human survival prediction. *Nat. Mach. Intell.* **1**, 95–104 (2019). 30801055 10.1038/s42256-019-0019-2 PMC6382062

[45] Marks, M. et al. Deep-learning-based identification, tracking, pose estimation and behaviour classification of interacting primates and mice in complex environments. *Nat. Mach. Intell.* **4**, 331–340 (2022). 35465076 10.1038/s42256-022-00477-5 PMC7612650

[46] Yang, L. et al. Machine learning for micro-and nanorobots. *Nat. Mach. Intell.* **6**, 605–618 (2024).

[47] LeCun, Y., Bengio, Y. & Hinton, G. Deep learning. *Nature* **521**, 436–444 (2015). 26017442 10.1038/nature14539

[48] Nikolenko, S. I. *Synthetic Data for Deep Learning*. Vol. 174 (Springer, 2021).

[49] Paproki, A., Salvado, O. & Fookes, C. Synthetic data for deep learning in computer vision & medical imaging: a means to reduce data bias. *ACM Comput. Surv.* **56**, 1–37 (2024).

[50] De Melo, C. M. et al. Next-generation deep learning based on simulators and synthetic data. *Trends Cogn. Sci.* **26**, 174–187 (2022). 34955426 10.1016/j.tics.2021.11.008

[51] Gao, C. et al. Synthetic data accelerates the development of generalizable learning-based algorithms for X-ray image analysis. *Nat. Mach. Intell.* **5**, 294–308 (2023). 38523605 10.1038/s42256-023-00629-1 PMC10959504

[52] Lu, Y. et al. Machine learning for synthetic data generation: a review. Preprint at https://arxiv.org/abs/2302.04062 (2023).

[53] Martyniak, S. et al. in *2025 IEEE/CVF Winter Conference on Applications of Computer Vision (WACV)* 4268–4278 (IEEE, 2025).

[54] Mohan, R., Arce, J., Mokhtar, S., Cattaneo, D. & Valada, A. Syn-Mediverse: a multimodal synthetic dataset for intelligent scene understanding of healthcare facilities. *IEEE Robot. Autom. Lett.* **9**, 7094–7101 (2024).

[55] Sarwin, G. et al. Patient-specific placental vessel segmentation with limited data. *J. Robot. Surg.* **18**, 237–245 (2024). 38833204 10.1007/s11701-024-01981-z PMC11150325

[56] Venkatesh, D. K., Rivoir, D., Pfeiffer, M., Kolbinger, F. & Speidel, S. In *2025 IEEE/CVF Winter Conference on Applications of Computer Vision (WACV)* 2280–2290 (IEEE).

[57] Zhang, Z., Yang, L. & Zheng, Y. In *2018 IEEE Conference on Computer Vision and Pattern Recognition (CVPR)* 9242–9251 (IEEE, 2018).

[58] Atapour-Abarghouei, A. & Breckon, T. P. In *2018 IEEE Conference on Computer Vision and Pattern Recognition (CVPR)* 2800–2810 (IEEE, 2018).

[59] Cheng, B., Saggu, I. S., Shah, R., Bansal, G. & Bharadia, D. In *2020**European Conference on Computer Vision (ECCV)* 52–69 (Springer, 2020).

[60] Zheng, C., Cham, T.-J. & Cai, J. in *2018 European Conference on Computer Vision (ECCV)* 767–783 (Springer, 2018).

[61] Ali, H., Murad, S. & Shah, Z. In *2022**Irish Conference on Artificial Intelligence and Cognitive Science (ICAICS)* 32–39 (Springer, 2022).

[62] Morís, D. I., Moura, J. D., Novo, J. & Ortega, M. Adapted generative latent diffusion models for accurate pathological analysis in chest X-ray images. *Med. Biol. Eng. Comput.* **62**, 2189–2212 (2024). 38499946 10.1007/s11517-024-03056-5 PMC11190015

[63] Packhäuser, K., Folle, L., Thamm, F. & Maier, A. In *2023 IEEE 20th International Symposium on Biomedical Imaging (ISBI)* 1–5 (IEEE).

[64] Sun, S., Goldgof, G., Butte, A. & Alaa, A. M. Aligning synthetic medical images with clinical knowledge using human feedback. *Adv. Neural Inf. Process. Syst.* **36**, 13408–13428 (2023).

[65] Akrout, M. et al. In *2023**International Conference on Medical Image Computing and Computer-Assisted Intervention (MICCAI),* 99–109 (Springer, 2023).

[66] Gonzales, A., Guruswamy, G. & Smith, S. R. Synthetic data in health care: a narrative review. *PLoS Digit. Health* **2**, e0000082 (2023). 36812604 10.1371/journal.pdig.0000082 PMC9931305

[67] Murtaza, H. et al. Synthetic data generation: state of the art in health care domain. *Compu. Sci. Rev.* **48**, 100546 (2023).

[68] Oh, H.-J. & Jeong, W.-K. In *2023**International Conference on Medical Image Computing and Computer-Assisted Intervention (MICCAI)* 337–345 (Springer, 2023).

[69] Veturi, Y. A. et al. SynthEye: investigating the impact of synthetic data on artificial intelligence-assisted gene diagnosis of inherited retinal disease. *Ophthalmol. Sci.* **3**, 100258 (2023). 36685715 10.1016/j.xops.2022.100258 PMC9852957

[70] Danielczuk, M., Angelova, A., Vanhoucke, V. & Goldberg, K. In *2020 IEEE/RSJ International Conference on Intelligent Robots and Systems (IROS)* 9577–9584 (IEEE).

[71] Wei, Y. et al. In *2020 ACM International Conference on Multimedia (ACM ICM)* 138–146 (ACM, 2020).

[72] Ward, T. M. et al. Computer vision in surgery. *Surgery* **169**, 1253–1256 (2021). 33272610 10.1016/j.surg.2020.10.039

[73] Kirillov, A. et al. In *2023 IEEE/CVF International Conference on Computer Vision (ICCV)* 4015–4026 (IEEE, 2023).

[74] Ma, J. et al. Segment anything in medical images. *Nat. Commun.* **15**, 654 (2024). 38253604 10.1038/s41467-024-44824-z PMC10803759

[75] Zhang, J., Huang, J., Jin, S. & Lu, S. Vision-language models for vision tasks: a survey. *IEEE Trans. Pattern Anal. Mach. Intell.* **46**, 5625–5644 (2024). 38408000 10.1109/TPAMI.2024.3369699

[76] Zhang, P. et al. In *2021 IEEE/CVF Conference on Computer Vision and Pattern Recognition (CVPR)* 5579–5588 (IEEE 2021).

[77] Mazurowski, M. A. et al. Segment anything model for medical image analysis: an experimental study. *Med. Image Anal.* **89**, 102918 (2023). 37595404 10.1016/j.media.2023.102918 PMC10528428

[78] Van, M. H., Verma, P. & Wu, X. In *2024**IEEE/ACM Conference on Connected Health: Applications, Systems and Engineering Technologies (CHASE*) 172–176 (IEEE).

[79] Wu, J. et al. Medical sam adapter: adapting segment anything model for medical image segmentation. *Med. Image Anal.* **102**, 103547 (2025). 40121809 10.1016/j.media.2025.103547

[80] Ye, J. et al. Loki: a comprehensive synthetic data detection benchmark using large multimodal models. Preprint at https://arxiv.org/abs/2410.09732 (2024).

[81] Azizi, S. et al. Robust and data-efficient generalization of self-supervised machine learning for diagnostic imaging. *Nat. Biomed. Eng.* **7**, 756–779 (2023). 37291435 10.1038/s41551-023-01049-7

[82] Ferreira, D. L., Lau, C., Salaymang, Z. & Arnaout, R. Self-supervised learning for label-free segmentation in cardiac ultrasound. *Nat. Commun.* **16**, 4070 (2025). 40307208 10.1038/s41467-025-59451-5 PMC12043926

[83] Krishnan, R., Rajpurkar, P. & Topol, E. J. Self-supervised learning in medicine and healthcare. *Nat. Biomed. Eng.* **6**, 1346–1352 (2022). 35953649 10.1038/s41551-022-00914-1

[84] Alabay, H. H., Le, T.-A. & Ceylan, H. X-ray fluoroscopy guided localization and steering of miniature robots using virtual reality enhancement. *Front. Robot. AI* **11**, 1495445 (2024). 39605865 10.3389/frobt.2024.1495445 PMC11599259

[85] Son, D., Ugurlu, M. C. & Sitti, M. Permanent magnet array–driven navigation of wireless millirobots inside soft tissues. *Sci. Adv.* **7**, eabi8932 (2021). 34669466 10.1126/sciadv.abi8932 PMC8528412

[86] Wu, S. et al. Magnetic milli-spinner for robotic endovascular surgery. *Adv. Mater.* **38**, e08180 (2025). 40970841 10.1002/adma.202508180 PMC12810593

[87] He, C. et al. Magnetically actuated dexterous tools for minimally invasive operation inside the brain. *Sci. Robot.* **10**, eadk4249 (2025). 40138483 10.1126/scirobotics.adk4249

[88] Mao, L. et al. Magnetic steering continuum robot for transluminal procedures with programmable shape and functionalities. *Nat. Commun.* **15**, 3759 (2024). 38704384 10.1038/s41467-024-48058-x PMC11069526

[89] Sun, M. et al. Exploiting ferrofluidic wetting for miniature soft machines. *Nat. Commun.* **13**, 7919 (2022). 36564394 10.1038/s41467-022-35646-y PMC9789085

[90] Ou, X. et al. Recent development in x-ray imaging technology: future and challenges. *Research* **2021**, 9892152 (2021). 35028585 10.34133/2021/9892152 PMC8724686

[91] Fan, X., Dong, X., Karacakol, A. C., Xie, H. & Sitti, M. Reconfigurable multifunctional ferrofluid droplet robots. *Proc. Natl Acad. Sci. USA* **117**, 27916–27926 (2020). 33106419 10.1073/pnas.2016388117 PMC7668164

[92] Sun, M. et al. Versatile, modular, and customizable magnetic solid-droplet systems. *Proc. Natl Acad. Sci. USA* **121**, e2405095121 (2024). 39088393 10.1073/pnas.2405095121 PMC11317579

[93] Brooks, T., Holynski, A. & Efros, A. A. In *2023 IEEE/CVF Conference on Computer Vision and Pattern Recognition (CVPR)* 18392–18402 (IEEE, 2023).

[94] Ozge Unel, F., Ozkalayci, B. O. & Cigla, C. In *2019 IEEE/CVF Conference on Computer Vision and Pattern Recognition (CVPR) Workshops* 582–591 (IEEE, 2019).

[95] Cheng, G. et al. Towards large-scale small object detection: survey and benchmarks. *IEEE Trans. Pattern Anal. Mach. Intell.* **45**, 13467–13488 (2023). 37384469 10.1109/TPAMI.2023.3290594

[96] Flores Duenas, C. A., Gaxiola Camacho, S. M. & Montaño Gómez, M. F. Radiographic dataset for VHS determination learning process. *Mendeley Data*10.17632/ktx4cj55pn.1 (2020).

[97] Jähne, B. *Digital Image Processing* (Springer, 2005).

[98] Szegedy, C., Vanhoucke, V., Ioffe, S., Shlens, J. & Wojna, Z. In *Proc. of the IEEE Conference on Computer Vision and Pattern Recognition* 2818–2826.

[99] Abdi, H. & Williams, L. J. Principal component analysis. *Wiley Interdiscip. Rev. Comput. Stat.* **2**, 433–459 (2010).

[100] Ultralytics YOLO11 v.11.0.0 (Ultralytics, 2024).

[101] Hein, D., Bozorgpour, A., Merhof, D. & Wang, G. Physics-inspired generative models in medical imaging: a review. Preprint at https://arxiv.org/abs/2407.10856 (2024). 10.1146/annurev-bioeng-102723-013922 40310888

[102] Walker, T. et al. in *2025 IEEE/CVF Conference on Computer Vision and Pattern Recognition (CVPR)* 1–15 (IEEE, 2025).

[103] van Rooij, W. J., Sprengers, M., de Gast, A. N., Peluso, J. & Sluzewski, M. 3D rotational angiography: the new gold standard in the detection of additional intracranial aneurysms. *Am. J. Neuroradiol.* **29**, 976–979 (2008). 18258703 10.3174/ajnr.A0964 PMC8128578

[104] Wang, C. et al. Daniosense: automated high-throughput quantification of zebrafish larvae group movement. *IEEE Trans. Autom. Sci. Eng.* **19**, 1058–1069 (2021).

[105] Ahmed, R., Ilami, M., Bant, J., Beigzadeh, B. & Marvi, H. A shapeshifting ferrofluidic robot. *Soft Robot.* **8**, 687–698 (2021). 33104417 10.1089/soro.2019.0184

[106] Li, J., Zhang, C., Zhu, W. & Ren, Y. A comprehensive survey of image generation models based on deep learning. *Ann. Data Sci.* **12**, 141–170 (2025).

[107] Kadambi, A., de Melo, C., Hsieh, C.-J., Srivastava, M. & Soatto, S. Incorporating physics into data-driven computer vision. *Nat. Mach. Intell.* **5**, 572–580 (2023).

[108] Sekh, A. A. et al. Physics-based machine learning for subcellular segmentation in living cells. *Nat. Mach. Intell.* **3**, 1071–1080 (2021).

[109] Zhang, H., Xu, H., Tian, X., Jiang, J. & Ma, J. Image fusion meets deep learning: a survey and perspective. *Inf. Fusion* **76**, 323–336 (2021).

[110] Azam, M. A. et al. A review on multimodal medical image fusion: compendious analysis of medical modalities, multimodal databases, fusion techniques and quality metrics. *Comput. Biol. Med.* **144**, 105253 (2022). 35245696 10.1016/j.compbiomed.2022.105253

[111] Tiryaki, M. E., Demir, S. O. & Sitti, M. Deep learning-based 3D magnetic microrobot tracking using 2D MR images. *IEEE Robot. Autom. Lett.* **7**, 6982–6989 (2023).

[112] Chen, F., Wang, X., Zhao, Y., Lv, S. & Niu, X. Visual object tracking: a survey. *Comput. Vis. Image Underst.* **222**, 103508 (2022).

[113] Yang, J. et al. Track anything: segment anything meets videos. Preprint at https://arxiv.org/abs/2304.11968 (2023).

[114] Hatamizadeh, A. et al. in *2022 IEEE/CVF Winter Conference on Applications of Computer Vision (WACV)* 574–584 (IEEE, 2022).

[115] Morimoto, T. et al. XR (extended reality: virtual reality, augmented reality, mixed reality) technology in spine medicine: status quo and quo vadis. *J. Clin. Med.* **11**, 470–479 (2022). 35054164 10.3390/jcm11020470 PMC8779726

[116] Bozkurt, A. & Sharma, R. C. Generative AI and prompt engineering: the art of whispering to let the genie out of the algorithmic world *AsianJDE* **18**, 1–7 (2023).

[117] Schumaker, L. *Spline Functions: Basic Theory* (Cambridge Univ. Press, 2007).

[118] Wang, C. X-ray-miniature-medical-device. *Hugging Face*10.57967/hf/7010 (2025).

[119] Cobo, A. M. *Parylene-Based**Implantable Interfaces for Biomedical Applications* (Univ. Southern California, 2017).

[120] Guo, H. et al. Wireless implantable optical probe for continuous monitoring of oxygen saturation in flaps and organ grafts. *Nat. Commun.* **13**, 3009 (2022). 35637230 10.1038/s41467-022-30594-z PMC9151749

[121] Sonmezoglu, S., Fineman, J. R., Maltepe, E. & Maharbiz, M. M. Monitoring deep-tissue oxygenation with a millimeter-scale ultrasonic implant. *Nat. Biotechnol.* **39**, 855–864 (2021). 33782610 10.1038/s41587-021-00866-y

