# Supplementary material for: Synthetic X‑ray‑driven tracking and control of miniature medical devices
Source: Nat Mach Intell. 2026 Feb 23;8(2):276–91. doi: 10.1038/s42256-026-01190-3 (PMC12932100; doi:10.1038/s42256-026-01190-3)
Supplement: Supplementary file 1 — Supplementary Figs. 1–15, Tables 1 and 2 and Notes for Supplementary Movies 1–8 [file 42256_2026_1190_MOESM1_ESM.pdf]

nature machine intelligence

Supplementary information https://doi.org/10.1038/s42256-026-01190-3

Synthetic X-ray-driven tracking and control of miniature medical devices

In the format provided by the authors and unedited

Supplementary Figures

Supplementary Fig. 1. Preparation of miniature medical devices data.
Supplementary Fig. 2. Schematic of the object localization algorithm.
Supplementary Fig. 3. Overview of the diffusion model training process.
Supplementary Fig. 4. Representative examples from the dataset.
Supplementary Fig. 5. Domain analysis.
Supplementary Fig. 6. Comparison between models trained with synthetic and real data.
Supplementary Fig. 7. Model performance after fine-tuning with real liquid MMD data.
Supplementary Fig. 8. Comparison between MicroSyn-X and clinical experts.
Supplementary Fig. 9. Image examples under different imaging conditions.
Supplementary Fig. 10. Hybrid robotic navigation strategy in live animals.
Supplementary Fig. 11. Evaluation of MicroSyn-X in environments of live animals.
Supplementary Fig. 12. Histological examination.
Supplementary Fig. 13. Tissue data for training diffusion models.
Supplementary Fig. 14. Quality assessment and parameter sensitivity in diffusion model inference.
Supplementary Fig. 15. Specifications of soft and liquid miniature medical devices.

Supplementary Tables

Supplementary Table 1. The locomotion data of soft MMDs.
Supplementary Table 2. The locomotion data of liquid MMDs.

Notes for Supplementary Movies

Supplementary Movie 1. Soft MMD tracking in diverse ex vivo tissue models.
Supplementary Movie 2. Soft MMD tracking in challenging imaging scenes.
Supplementary Movie 3. Liquid MMD tracking in diverse ex vivo tissue models.
Supplementary Movie 4. Liquid MMD tracking in challenging imaging scenes.
Supplementary Movie 5. Soft MMD deployment in 3D porcine arteries.
Supplementary Movie 6. Multi-MMD deployment in the ex vivo tissue model.
Supplementary Movie 7. Soft MMD deployment in the rabbit femoral arterial network in vivo.
Supplementary Movie 8. Soft MMD deployment in rat arterial regions in vivo.

Supplementary Figures

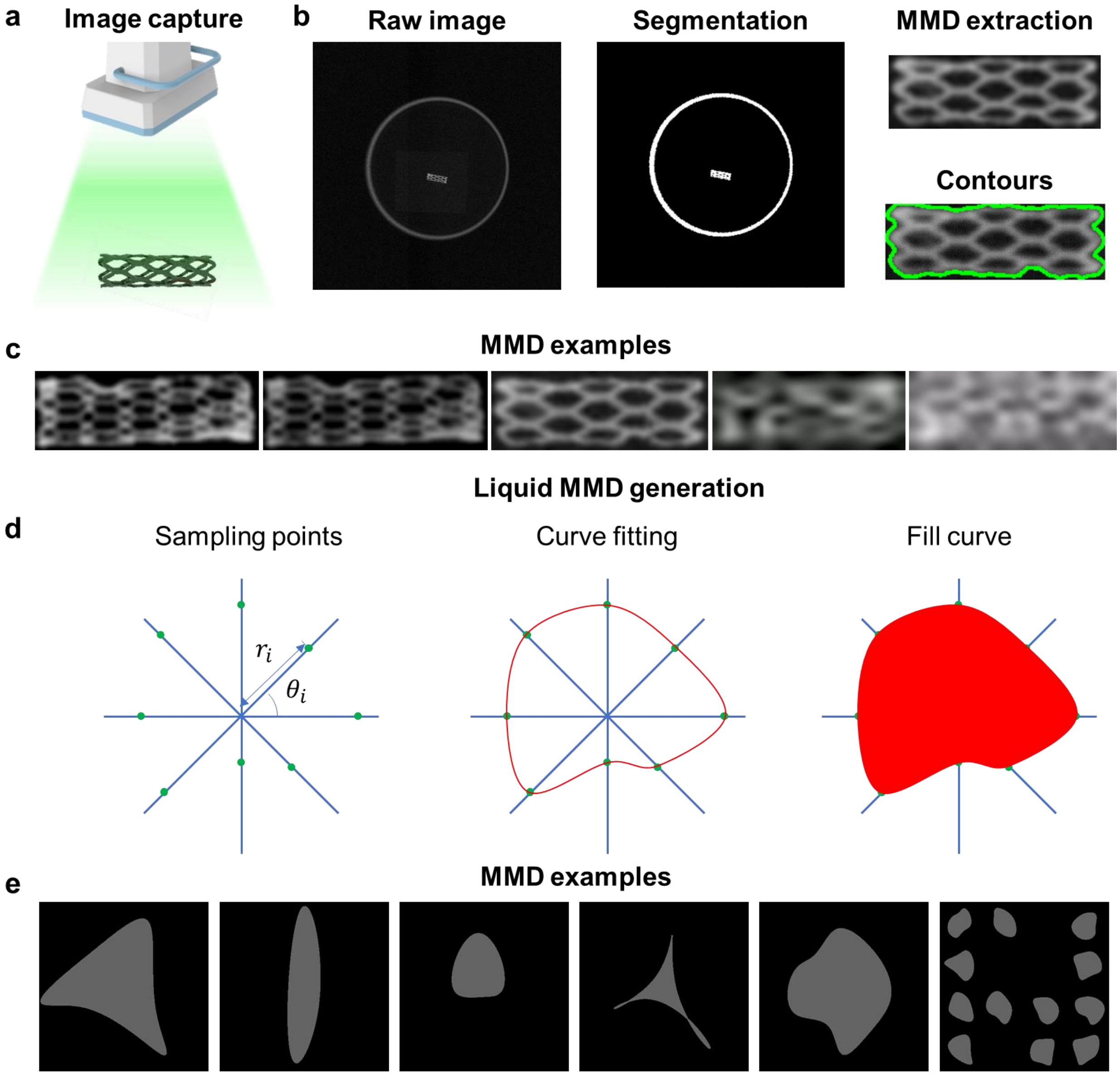

Supplementary Fig. 1. Preparation of miniature medical devices data. a. Image capture under X-ray imaging. **b.** Extraction of MMD images. For image extraction, the MMD is positioned in a petri dish with a clean background. Automatic segmentation is performed using Otsu method, yielding a pixel-accurate contour for subsequent cropping and rotation. **c.** Representative examples of captured MMD images. **d.** Workflow for generating synthetic liquid MMD images. **e.** Representative examples of generated MMD images.

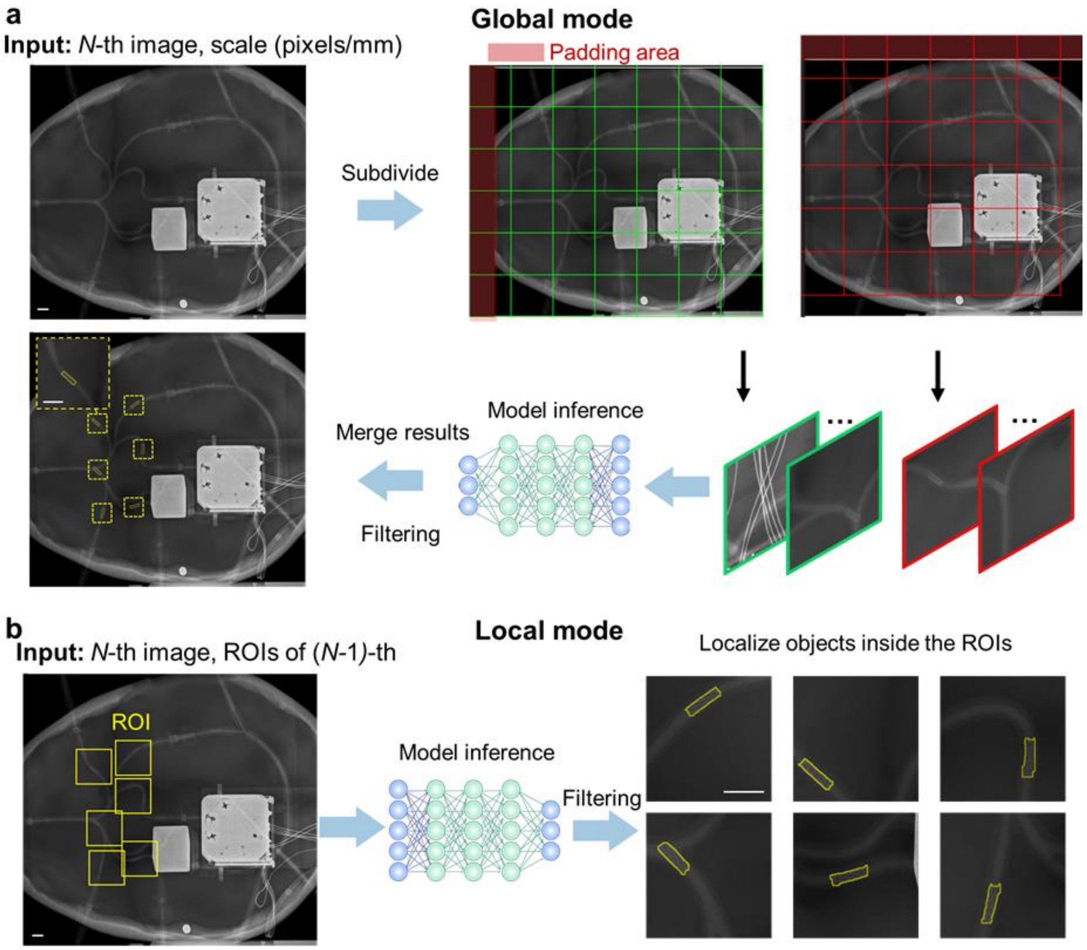

Supplementary Fig. 2. Schematic of the object localization algorithm. a. Schematics of the global mode. The input image is partitioned into overlapping patches, where patch size is adaptively determined based on image scale (pixels/mm). Each patch is processed independently by the model, and the resulting detections are aggregated and merged to produce the final localization output. **b.** Schematics of the local mode. Leveraging prior localization results, the algorithm focuses computation on regions of interest (ROIs), feeding only these refined subregions into the model to enhance the efficiency. In all figures, scale bars represent 5 mm.

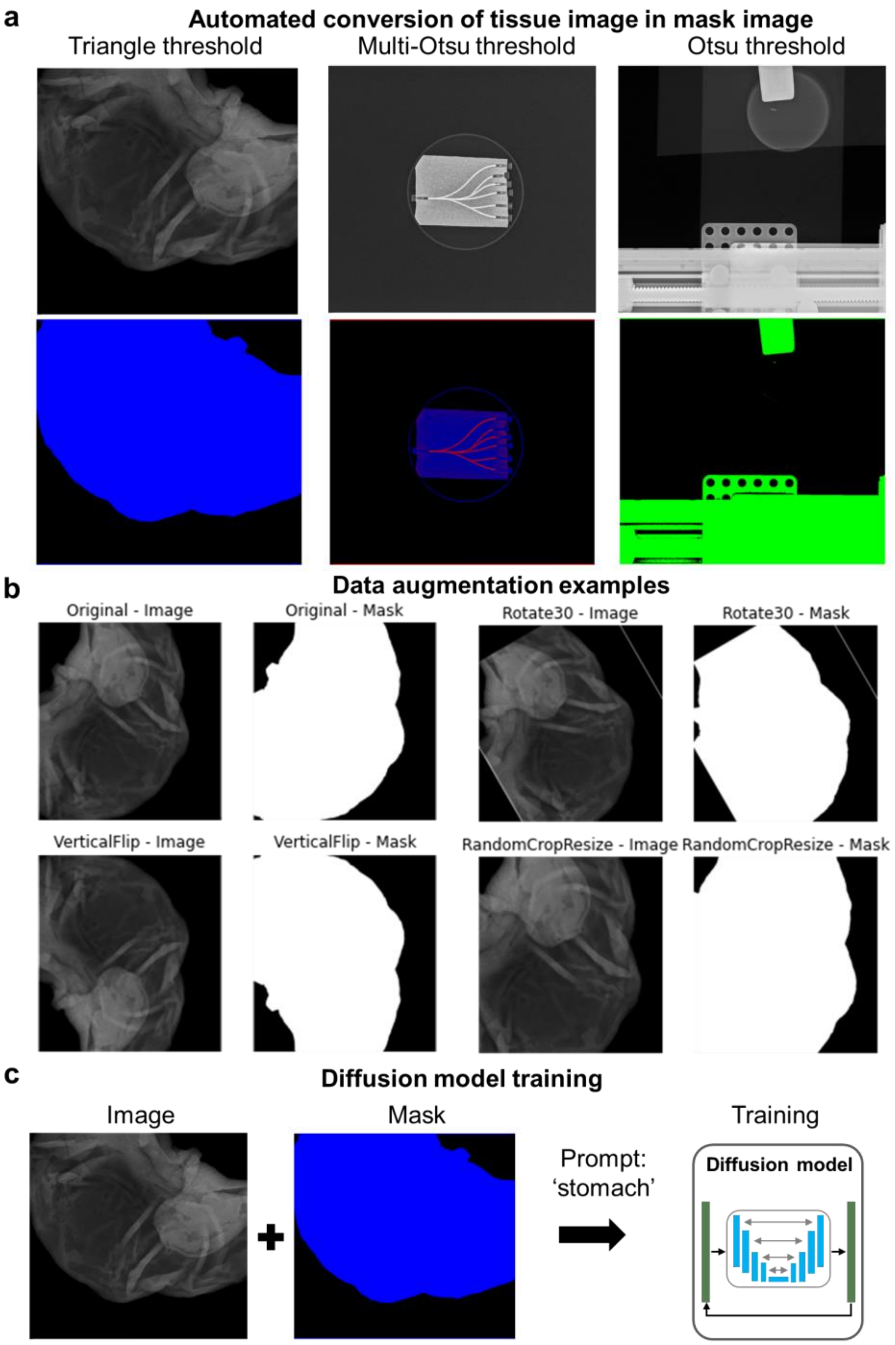

Supplementary Fig. 3. Overview of the diffusion model training process. a. Automated Image Segmentation. The image is automatically segmented into mask image, composed of three channels (blue for tissue area $\mathbf{M}_{\text{tissue}}$, green for metallic device area $\mathbf{M}_{\text{device}}$, and red for lumens with contrast agent area $\mathbf{M}_{\text{lumen}}$). Different thresholding methods are applied to perform the segmentation. **b.** Data augmentation. To increase dataset diversity and improve the ability of the model to learn texture features, data augmentation techniques are applied, including geometric transformations and color-space modifications. **c.** Diffusion Model Training. The segmented mask, original image, and corresponding prompt are used to train the diffusion model.

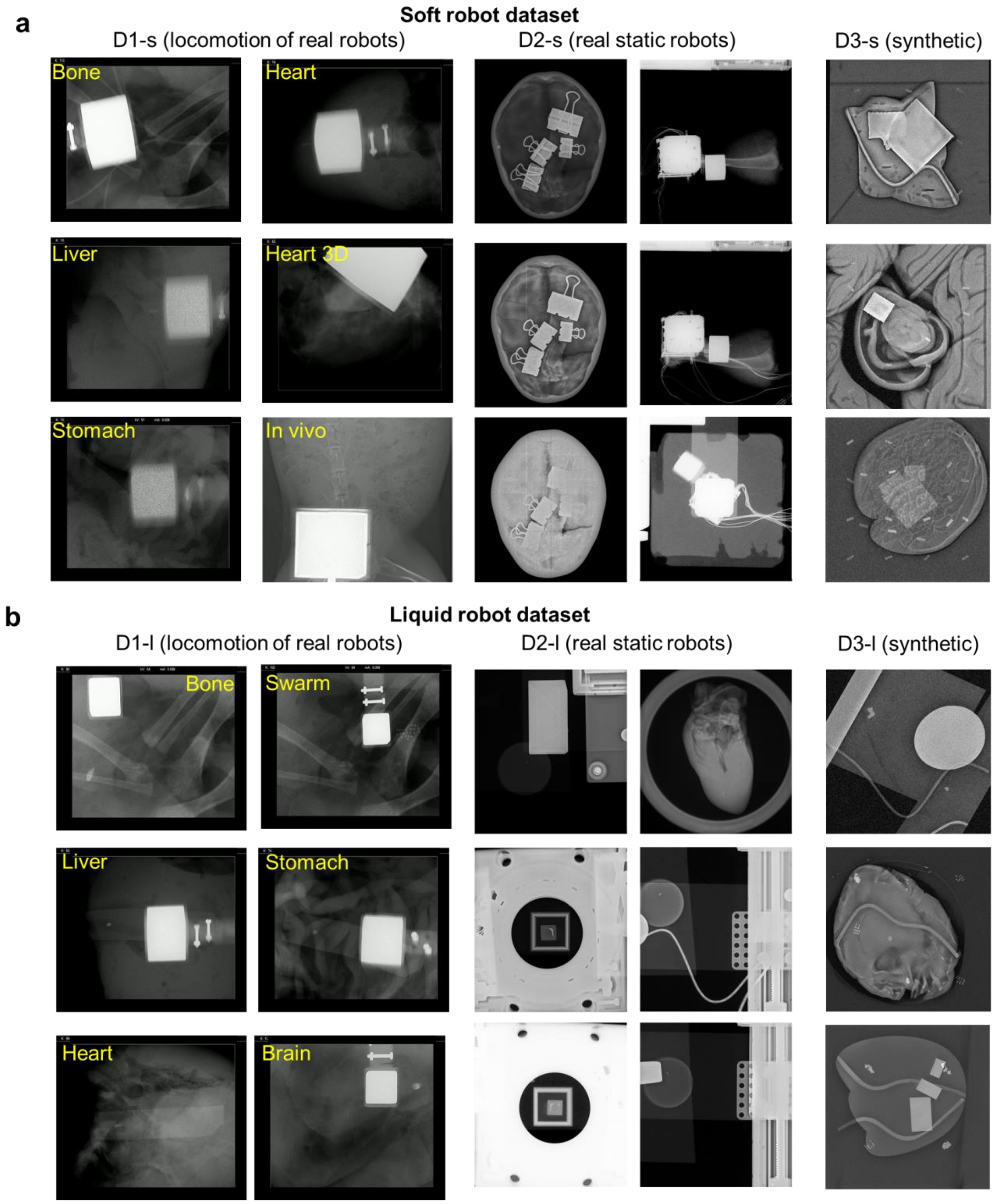

Supplementary Fig. 4. Representative examples from the dataset.

a

Algorithm 1 Feature Space Coverage Computation between Dataset 1 and Dataset 2

Require: Image paths from dataset 1: $\mathcal{I}_1$; Image paths from dataset 2: $\mathcal{I}_2$; Pretrained model $\mathcal{M}$; Percentile thresholds $\mathcal{P}$

Ensure: Coverage scores and radii for each percentile in $\mathcal{P}$

Initialize empty feature sets $\mathcal{F}_1 \leftarrow \emptyset$, $\mathcal{F}_2 \leftarrow \emptyset$
$\triangleright$ Extract features from Dataset 1
for each mini-batch $B \subset \mathcal{I}_1$ **do**
    Load and preprocess images in B
    Extract features using model $\mathcal{M}$
    Flatten and append to $\mathcal{F}_1$
end for
$\triangleright$ Extract features from Dataset 2
for each mini-batch $B \subset \mathcal{I}_2$ **do**
    Load and preprocess images in B
    Extract features using model $\mathcal{M}$
    Flatten and append to $\mathcal{F}_2$
end for
for each percentile $p \in \mathcal{P}$ **do** $\triangleright$ Step 1: Estimate local neighborhood density in Dataset 1
    Fit a 2-Nearest Neighbors (2-NN) model on $\mathcal{F}_1$
    For each feature vector $f \in \mathcal{F}_1$, compute distance to its 2nd nearest neighbor (excluding itself)
    Collect all such distances into array D_1
    $\triangleright$ Step 2: Define a coverage radius
    Compute coverage radius $r_p \leftarrow$ the pth percentile of D_1
    $\triangleright$ Step 3: Measure coverage by Dataset 2
    Fit a 1-Nearest Neighbor (1-NN) model using features $\mathcal{F}_2$
    For each $f \in \mathcal{F}_1$, compute distance to its nearest neighbor in $\mathcal{F}_2$
    Count number of feature vectors from $\mathcal{F}_1$ with nearest neighbor distance $\leq r_p$, store in `covered`
    $\triangleright$ Step 4: Compute and store the coverage score
    $C_p \leftarrow \frac{\texttt{covered}}{|\mathcal{F}_1|} \times 100$
    Store (C_p, r_p)
end for

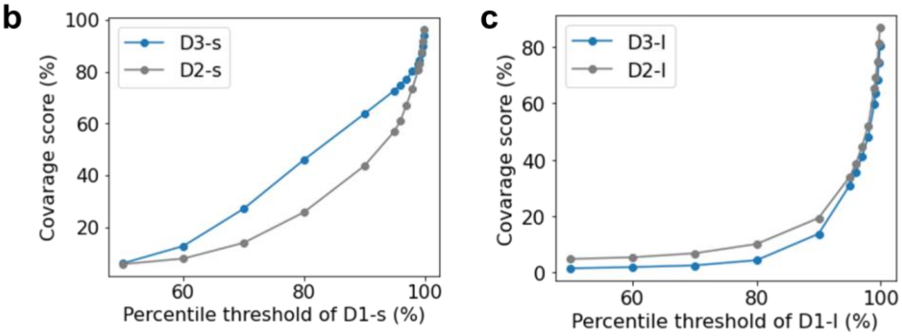

Supplementary Fig. 5. Domain analysis. a. Dataset coverage analysis workflow. The backbone of the convolutional neural network trained with synthetic data was used to extract features. Percentile thresholds are defined as a percentage of the maximum intra-class feature distance and are used to compute the coverage ratio. **b.** Data coverage results. D1 represents the test dataset containing MMD locomotion in various tissue environments. D2 consists of static MMD images, and D3 contains synthetic MMD images. The symbols s and l denote soft MMDs and liquid MMDs, respectively.

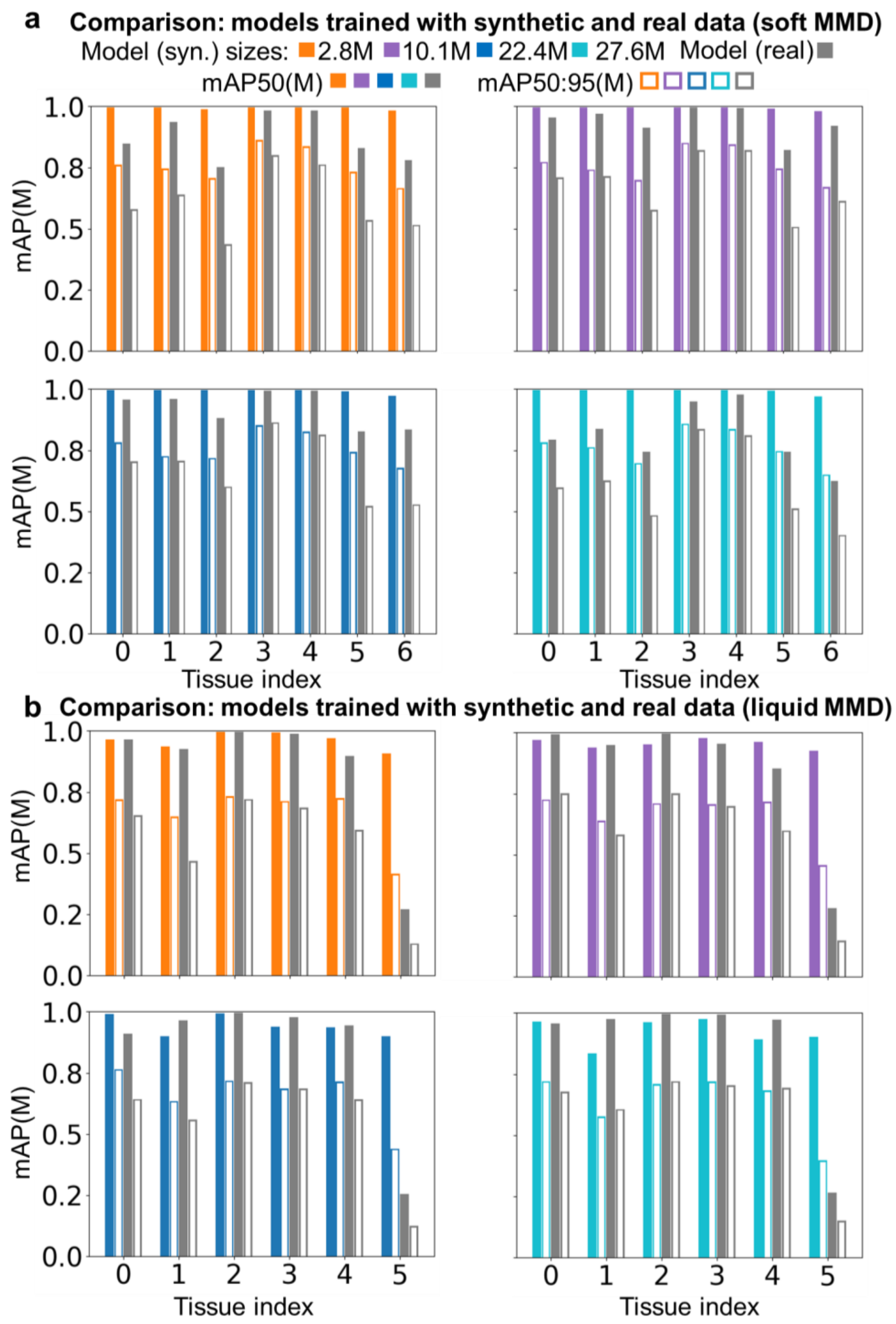

Supplementary Fig. 6. Comparison between models trained with synthetic and real data. a. Performance of models trained on synthetic and real soft MMD data, measured by mAP50(M), and mAP50:95(M). Tissue index indicates datasets: soft MMDs in porcine brain with embedded bones, porcine brain, heart, liver, stomach, heart 3D vessels, and in vivo animals. **b.** Performance of models trained on synthetic and real liquid MMD data. Tissue index indicates datasets: liquid MMDs in porcine brain, heart, liver, stomach, brain or liver with embedded bones, and MMD swarm under bone occlusion.

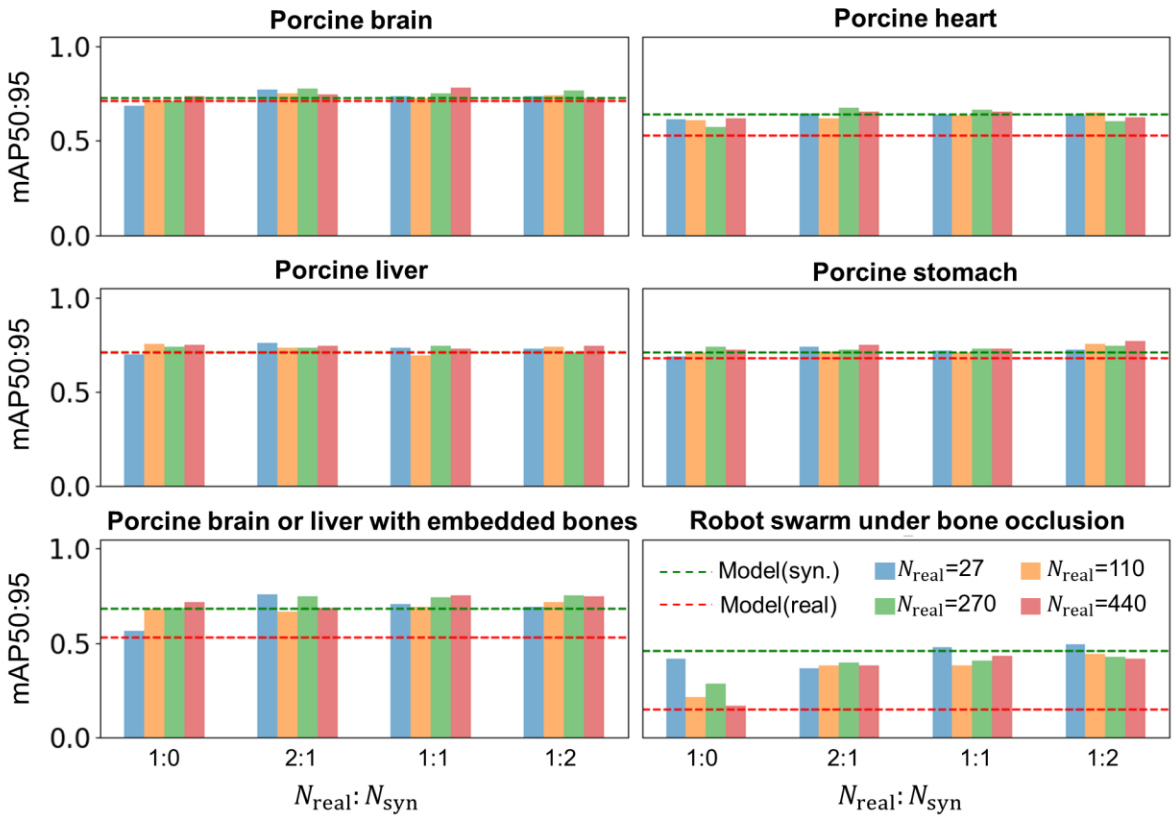

Supplementary Fig. 7. Model performance after fine-tuning with real liquid MMD data. Different numbers of real images are incorporated for fine-tuning. Simple fine-tuning using only real data leads to imbalanced performance—improving classes well represented in the real dataset while degrading underrepresented ones. In contrast, balanced fine-tuning with both synthetic and real data preserves previously learned knowledge while enabling adaptation to the real domain. N_{real} and N_{syn} denote the numbers of real and synthetic images, respectively. The model with 10.1M parameters is used in this analysis. The green and red dashed lines represent the results of model trained on synthetic images of D1-l and real images of D2-l, respectively.

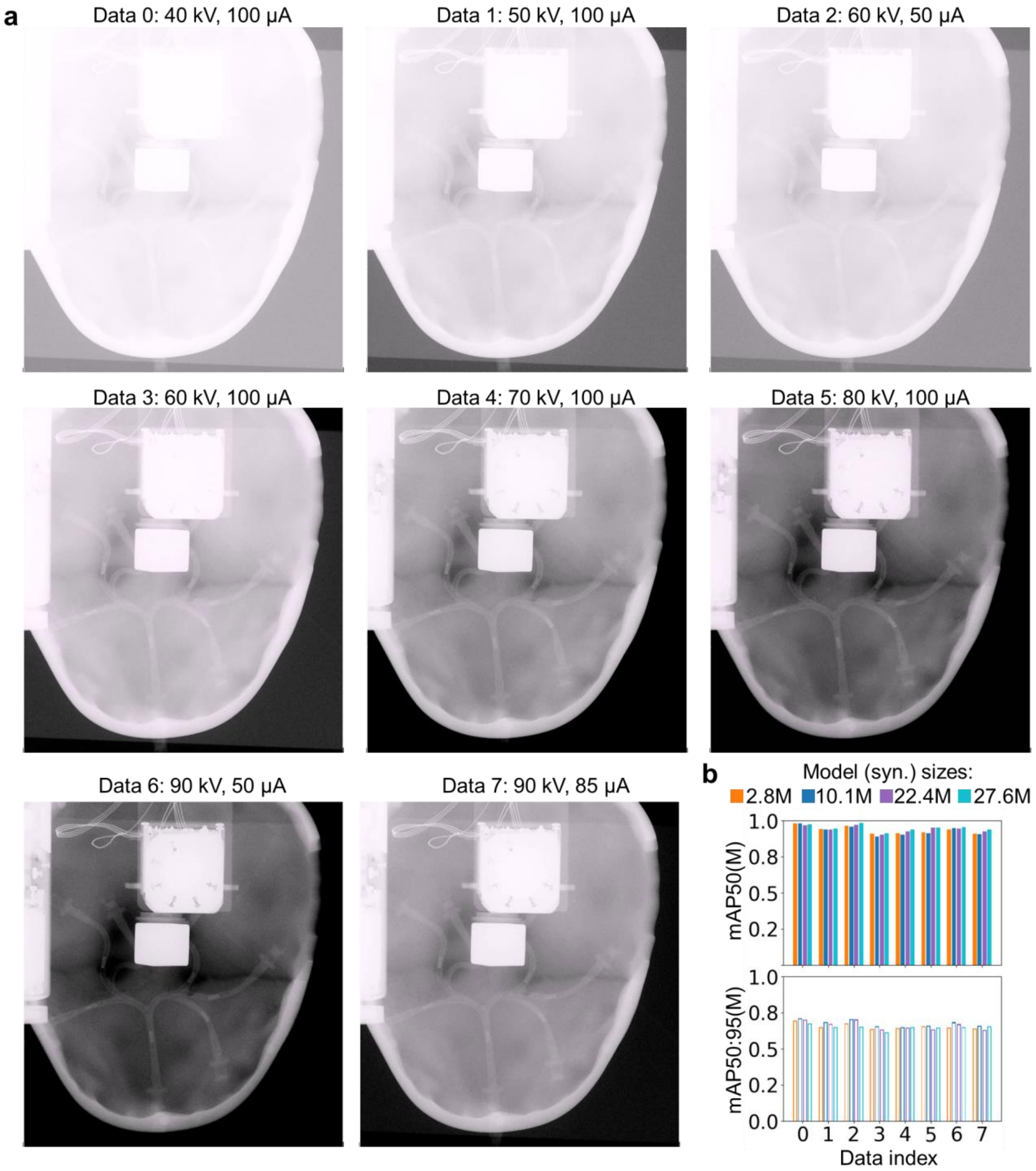

Supplementary Fig. 8. Comparison between MicroSyn-X and clinical experts. a. Images for comparison between models trained with synthetic data (model (syn.)) and manual annotation. **b.** Performance evaluation of models in detecting MMDs that are manually identifiable by clinical experts. Models of different sizes trained on synthetic data were evaluated.

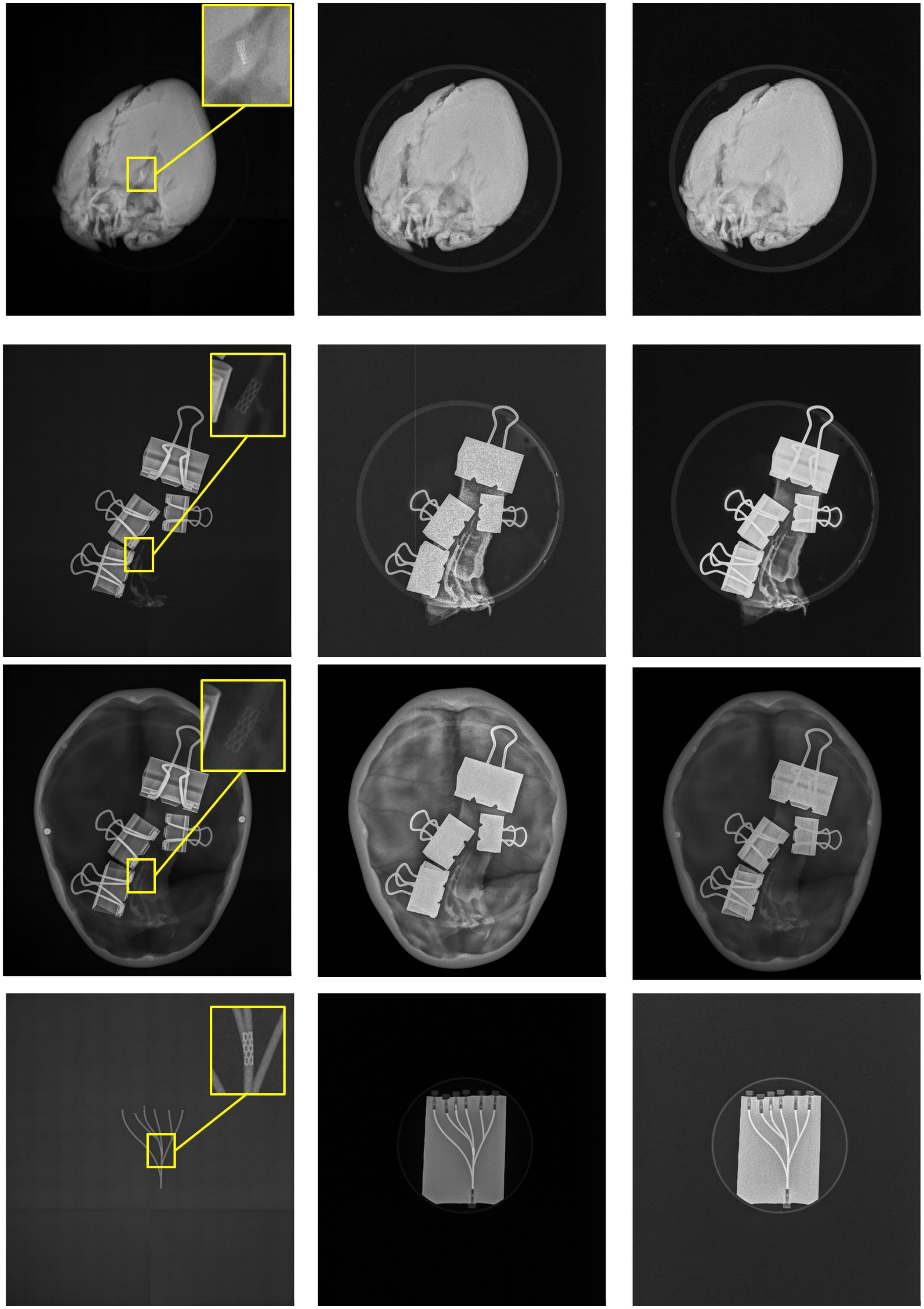

Supplementary Fig. 9. Image examples under different imaging conditions.

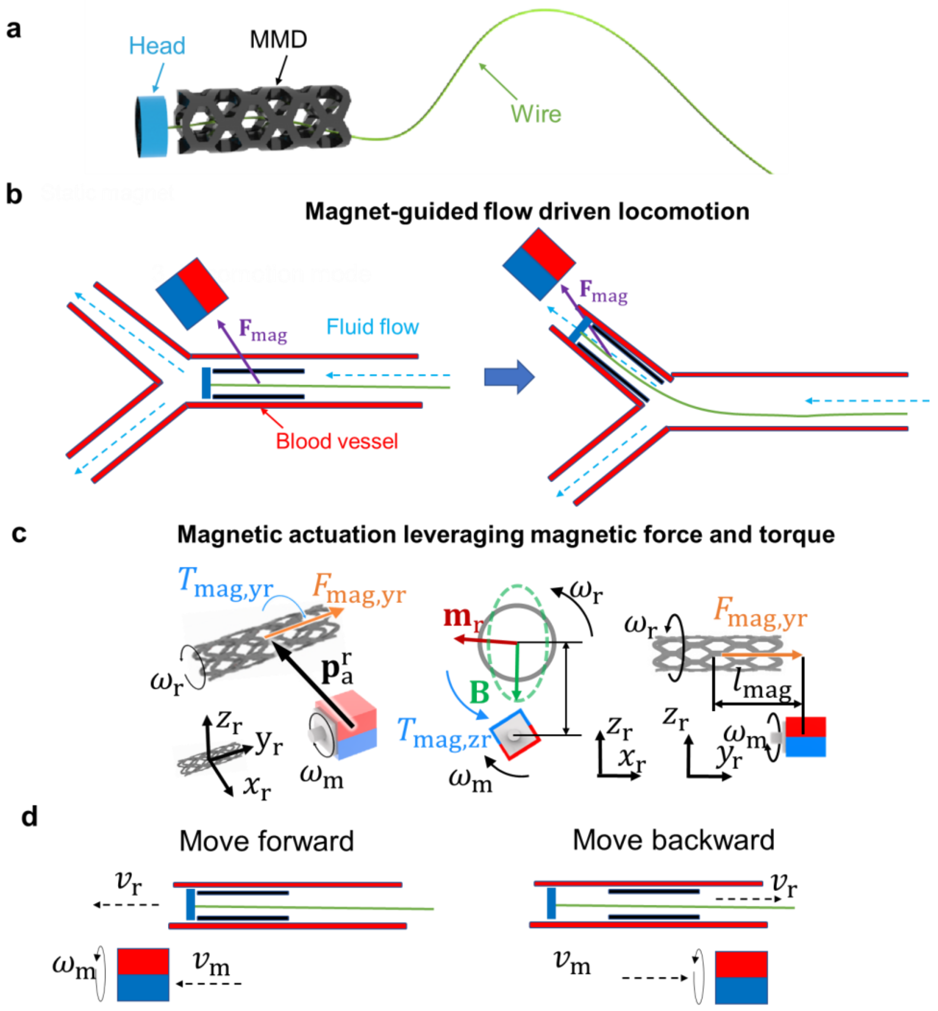

Supplementary Fig. 10. Hybrid robotic navigation strategy in live animals. a. Device for MMD deployment in in vivo environment. A surgical monofilament suture (Ethilon 8-0, Ethicon Co. Ltd.) attached to a 3D printed head (IPQ, Nanoscribe GmbH) functions as a safety cable to enable flow-driven locomotion and ensure fail-safe control during clinical interventions. **b.** Magnet-guided flow driven locomotion. The MMD is driven by fluid flow to the desired branch, with its direction guided by a static magnetic field. **c.** Magnetic actuation using a rotating permanent magnet. Magnetic torque and force are utilized to actuate the MMD. **d.** MMD locomotion under magnetic actuation.

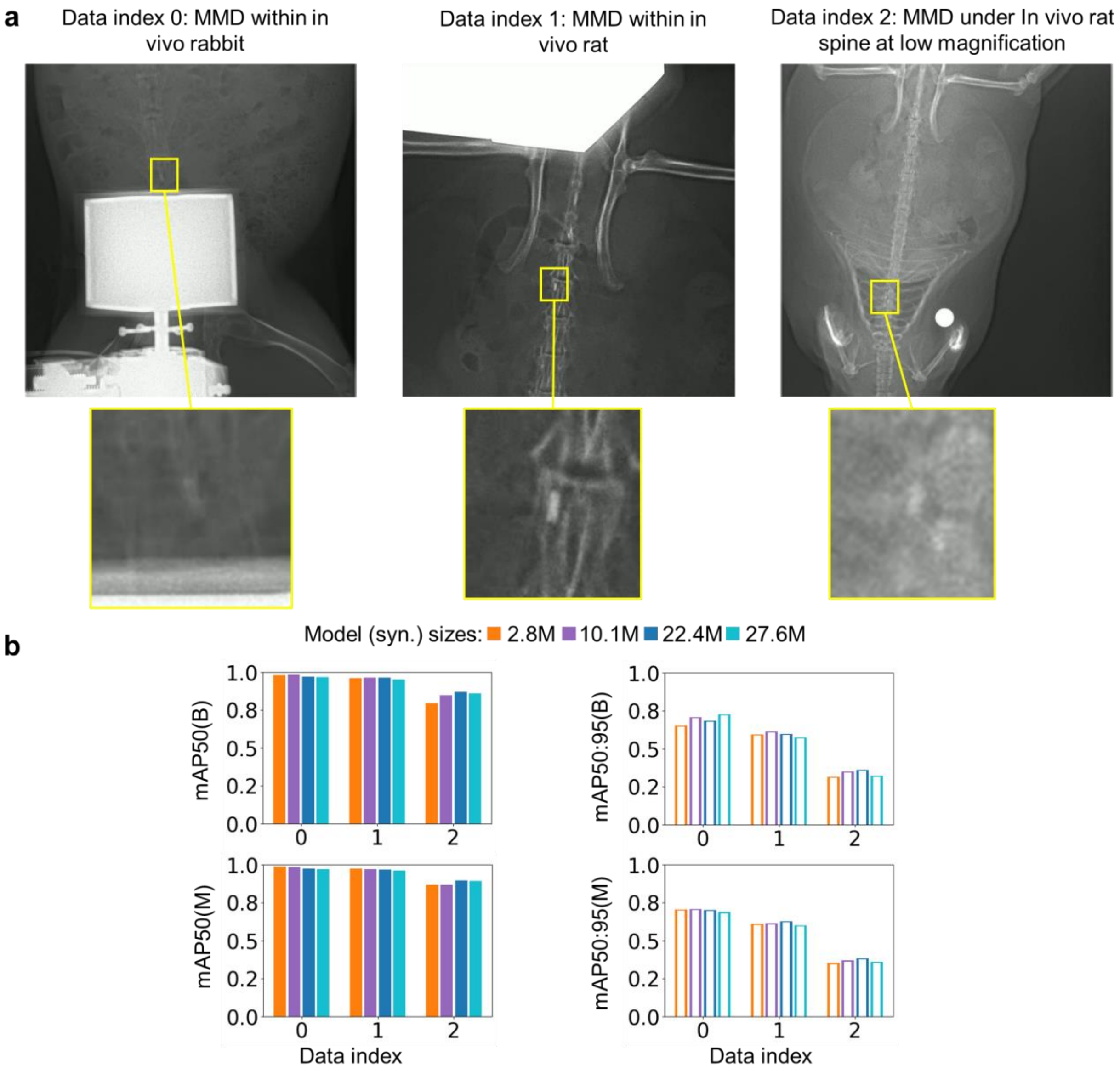

Supplementary Fig. 11. Evaluation of MicroSyn-X in environments of live animals. a. Dataset of in vivo MMD navigation. For data 2 (stress test scenario), the MMD appeared at low resolution and was heavily occluded by the rat spine. b. Model performance on in vivo datasets. Models trained on synthetic data were evaluated on in vivo scenarios.

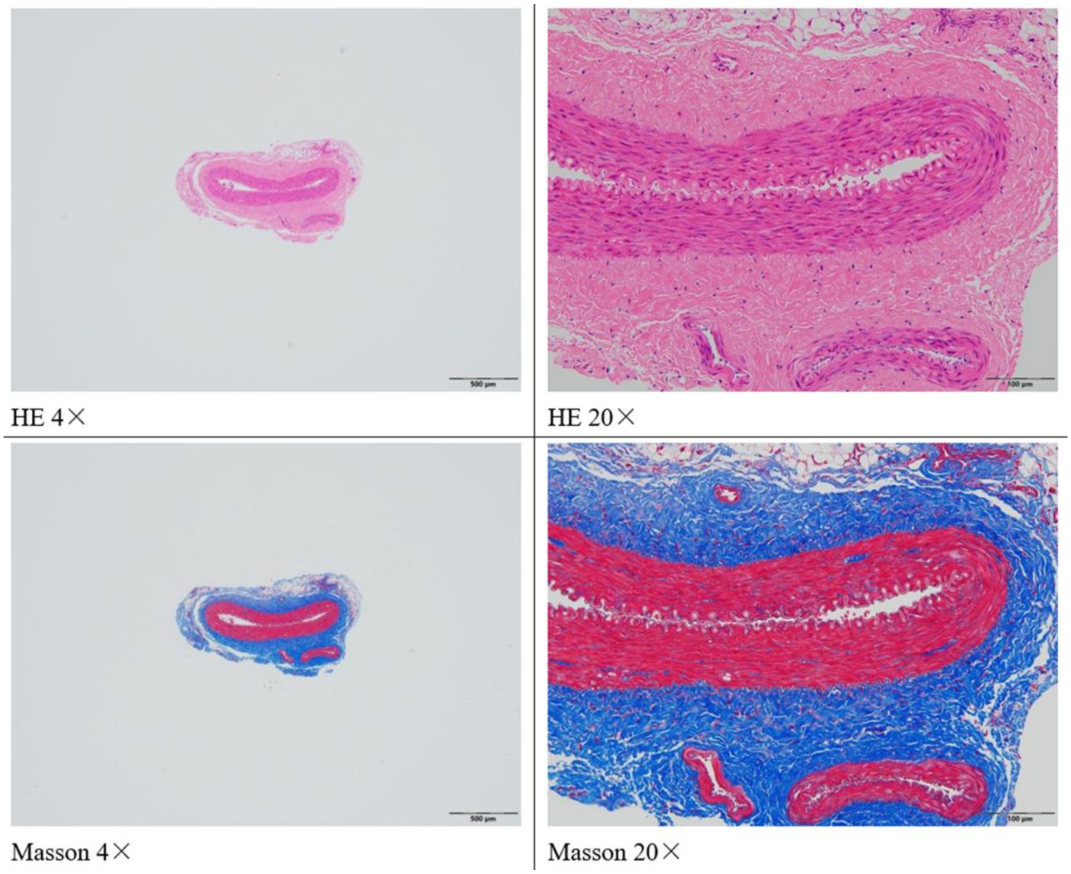

Supplementary Fig. 12. Histological examination. The histological images showed that the vascular lumen was open with no evidence of obstruction or thrombus formation. Endothelial cells of the vessel wall were arranged regularly, with no signs of hyperplasia or detachment. The internal elastic lamina was intact, with no signs of loss or rupture. The tunica media was composed of circumferentially arranged smooth muscle cells, showing no damage. The tunica adventitia consisted of loose connective tissue and appears undamaged. Masson's trichrome staining revealed no significant fibrous tissue proliferation in the intima, media, or adventitia of the vessel wall.

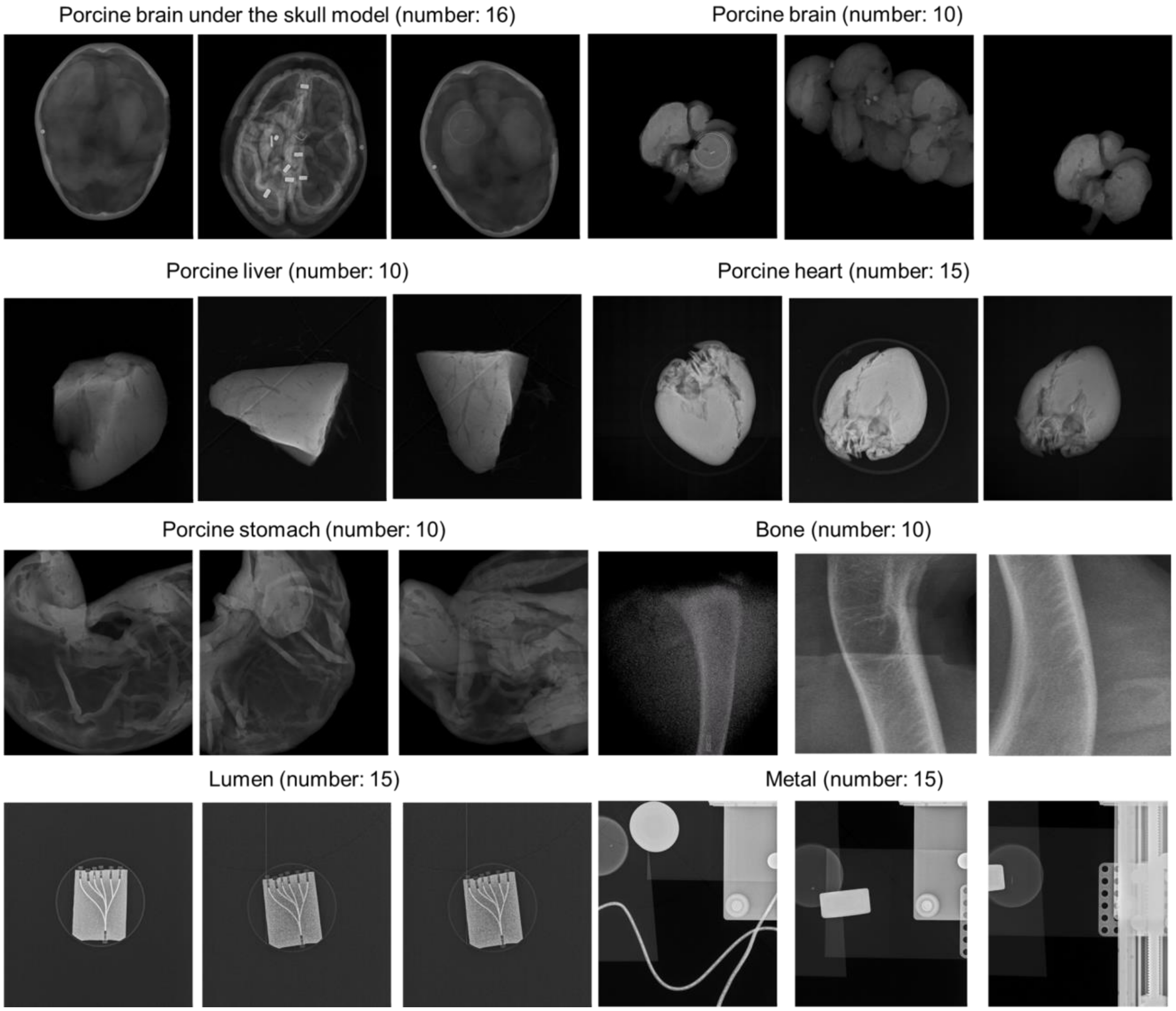

Supplementary Fig. 13. Tissue data for training diffusion models.

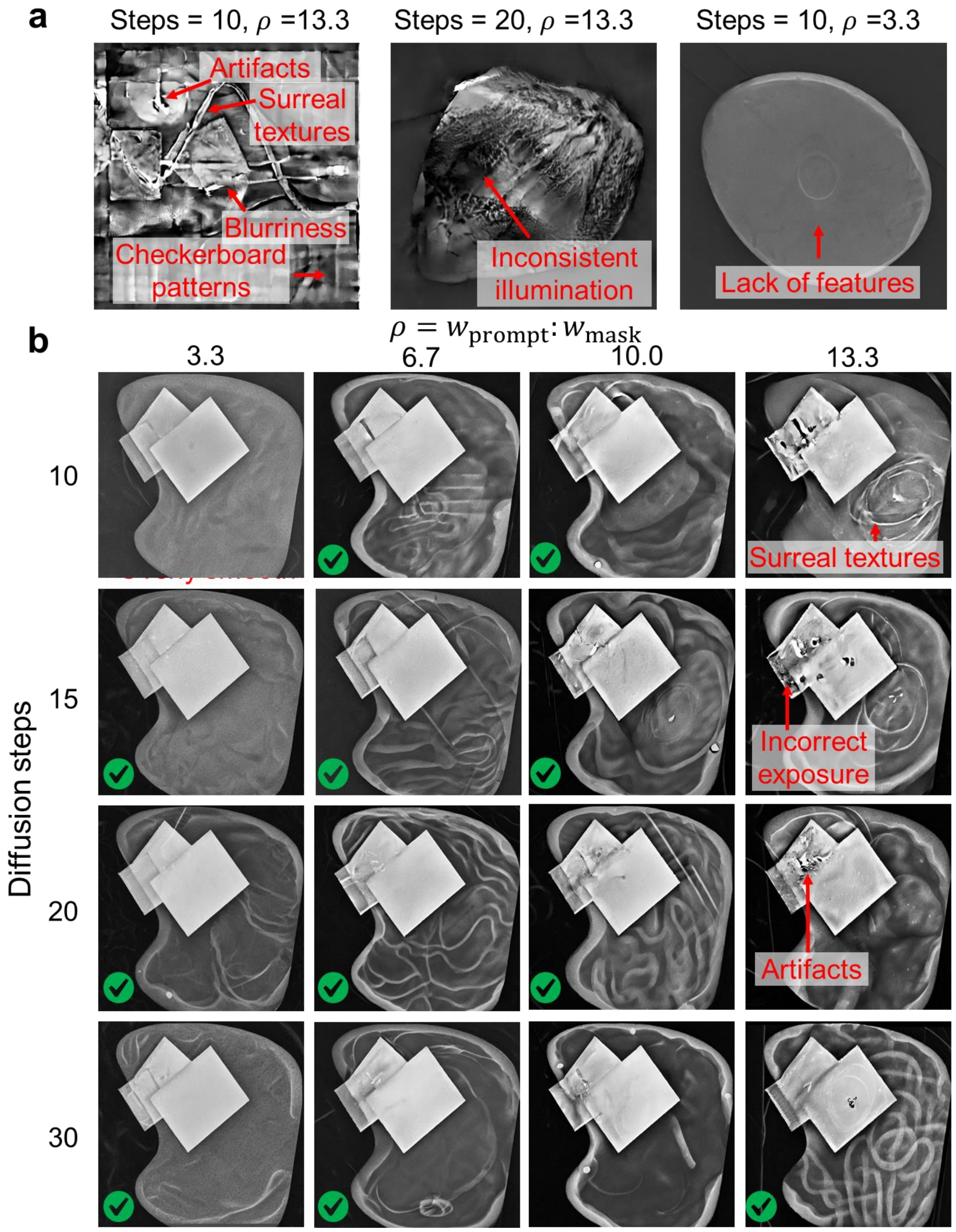

Supplementary Fig. 14. Quality assessment and parameter sensitivity in diffusion model inference. a. Characteristic artifacts in low-quality generations, categorized as: generation artifacts (surreal textures, blurriness, high-frequency noise, grid/checkerboard patterns), physical inconsistencies (non-uniform illumination, exposure mismatches, implausible contrast), and texture deficiencies (excessive smoothness in structurally detailed regions). **b.** Trade-offs between number of diffusion steps and classifier-free guidance scale: low diffusion step counts combined with high prompt guidance weights are prone to produce unstable or distorted outputs. Parameters selected for final inference are indicated by green markers.

Soft MMD specifications

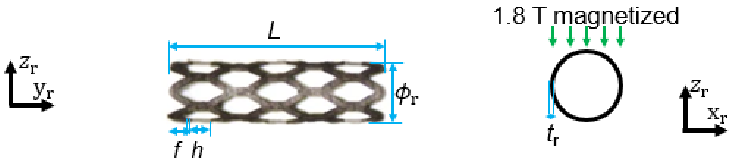

Parameters	Values (design 1)	Values (design 2)
Strict spacing *h* (mm)	0.40	0.20
Axial amplitude of each segment *f* (mm)	0.50	0.25
Overall length *L* (mm)	5.00	1.85
Initial diameter ϕ_r (mm)	1.50	0.55
Wall thickness t_r (mm)	0.10	0.10
PDMS (mass ratio)	10:1	10:1
NdFeB:PDMS (mass ratio)	4:1	4:1

Liquid MMD specifications

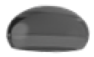

Parameters	Values
Density (g/cm^3)	1.43
Dynamic viscosity (mPa·s)	8.00
Volume (μL)	40-60

Supplementary Fig. 15. Specifications of soft and liquid miniature medical devices.

Tissue	Imaging parameters (kV/μA)	Locomotion distance (mm)	Mean robot Speed (mm/s)	Magnet rotation rate (Hz)	Localization success rate
Bone (15-25 mm), porcine liver	61/97	79.12	0.41	0.3-0.7	98.4%
Porcine heart	60/56	68.63	0.42	0.3-0.7	57.4%
	66/97	51.66	0.68	0.9-1.3	23.4%
Porcine liver	53/64	48.85	0.40	0.3-0.7	98.8%
Porcine stomach	51/97	60.03	0.33	0.3-0.7	99.6%
Bone (5-15 mm), porcine brain, skull model	56/97	67.52	0.28	0-0.7	78.4%
	56/97	42.55	0.36	0-0.7	71.9%
	56/97	32.93	0.24	0-0.7	73.7%
	56/97	22.41	0.20	0-0.7	81.9%
Porcine heart artery, skull model	73/97	83.23	0.30	0-0.7	80.0%
Porcine brain, skull model	54/98	31.56	0.21	0.3-0.7	95.1%
	57/97	74.84	0.43	0.3-0.7	98.6%
	57/97	75.95	0.38	0.3-0.7	99.1%
	57/97	53.18	0.29	0.3-0.7	95.5%
Rabbit femoral arteries in vivo	57/98	94.91	0.53	0-1.0	93.8%
	57/98	49.39	0.38	0-1.0	94.5%
	57/98	47.38	0.44	0-1.0	91.7%
Rat aorta in vivo	58/86	76.52	2.59	0	42.4%
	58/86	26.66	1.71	0	78.3%
Rat lilac artery in vivo	58/86	9.45	0.21	0-1.0	83.7%

Supplementary Table 1. The locomotion data of soft MMDs.

Tissue	Imaging parameters (kV/μA)	Locomotion distance (mm)	Mean robot speed (mm/s)	Magnet rotation rate (Hz)	Localization success rate
Porcine stomach	51/97	29.47	0.28	0-0.3	65.4%
Porcine brain, skull model	51/60	28.92	0.38	0-0.3	97.4%
Porcine liver	54/97	33.32	0.39	0-0.3	98.9%
Porcine heart	55/68	20.95	0.17	0-0.3	52.6%
Bone (15-25 mm), porcine liver	60/98	25.25	0.20	0-0.3	46.6%
Bone (5-15 mm), porcine brain, skull model	54/97	204.95	0.55	0	96.6%
	54/97	30.62	0.73	0	74.6%

Supplementary Table 2. The locomotion data of liquid MMDs.

Supplementary Movies

Supplementary Movie 1. Soft MMD tracking in diverse ex vivo tissue models.
This video demonstrates the navigation and tracking performance of stent-structured MMDs across various ex vivo biological tissues. The tracking capability was validated under challenging conditions, including bone with thicknesses up to 25 mm, as well as porcine heart, liver, stomach, and brain tissues. Despite the diverse tissue textures, imaging noise, and partial occlusions, the algorithm maintained robust and accurate tracking throughout the experiments.

Supplementary Movie 2. Soft MMD tracking in challenging imaging scenes.
This video illustrates the navigation and tracking performance of stent-structured MMDs within a skull phantom featuring randomly placed bones to simulate dense anatomical obstructions. A soft MMD navigated through a contrast-agent-filled lumen, successfully traversing bifurcations and reversing direction despite persistent occlusions caused by bone structures. Additionally, the robustness of the tracking system was validated under frequent mechanical occlusions and degraded imaging quality.

Supplementary Movie 3. Liquid MMD tracking in diverse ex vivo tissue models.
This video showcases the navigation and tracking performance of ferrofluid-based MMDs across a range of ex vivo biological tissues. The tracking system was validated under challenging conditions, including bone with thicknesses up to 25 mm, as well as porcine heart, liver, stomach, and brain tissues. Despite variations in tissue texture, imaging noise, partial occlusions, and changes in MMD morphology, the MMDs were robustly tracked throughout the experiments.

Supplementary Movie 4. Liquid MMD tracking in challenging imaging scenes.
This video presents the navigation and tracking of a ferrofluid-based MMD within an MPI-shaped structure containing randomized bone occlusions. The MMD underwent significant deformation to traverse narrow channels while remaining continuously tracked. Additionally, dynamic behaviours such as the splitting and merging of swarm-like ferrofluid formations were tracked throughout the merging process, despite persistent occlusions from bone structures and external magnets.

Supplementary Movie 5. Soft MMD deployment in 3D porcine arteries.
This video demonstrates the deployment of a stent-structured MMD within a 3D-shaped ex vivo blood vessel, visualized using contrast-enhanced C-arm imaging from three orthogonal angles. A 3D vascular path was reconstructed from the contrast agent-filled lumen, providing an anatomical roadmap for navigation. Subsequently, a planning algorithm computed magnet trajectories for magnetic actuation within spatial constraints. The MMD followed user commands along the planned route, guided by real-time tracking, and the C-arm angle was adjusted to maintain visibility. Despite imaging noise, low contrast, partial occlusions, and MMD 3D rotation, the MMD is continuously and accurately tracked.

Supplementary Movie 6. Multi-MMD deployment in the ex vivo tissue model.
This video demonstrates the system scalability through multi-MMD validation. Three soft MMDs were sequentially deployed and simultaneously tracked in separate lumens beneath a porcine brain and skull model, with robust tracking maintained despite occlusions.

Supplementary Movie 7. Soft MMD deployment in the rabbit femoral arterial network in vivo.
This video presents an in vivo demonstration of a soft MMD navigating the rabbit femoral arterial network, successfully traversing four predefined waypoints and multiple branches. Utilizing rotating magnetic actuation, the MMD entered two branches and performed bidirectional locomotion.

Supplementary Movie 8. Soft MMD deployment in rat arterial regions in vivo.
This video demonstrates a rat model in which the MMD was delivered into the abdominal aorta and carried by blood flow to a predefined waypoint before entering a bifurcation. Despite a large imaging window, low resolution, and continuous spinal occlusion, the tracking algorithm maintained effective localization. Leveraging combined magnetic guidance and fluid dynamics, the MMD successfully navigated the bifurcation, after which a rotating permanent magnet actuated it toward the distal target area.
